# Au(I)‐, Au(II)‐, Au(III)‐Fluoride Complexes: Synthesis and Applications in Organic Transformations

**DOI:** 10.1002/anie.202424656

**Published:** 2025-02-26

**Authors:** Alexi T. Sedikides, Rhian C. Walters, Alice C. Dean, Alastair J. J. Lennox

**Affiliations:** ^1^ School of Chemistry University of Bristol Bristol BS8 1TS UK

**Keywords:** gold, oxidation, catalysis, organometallics, fluorine

## Abstract

The synthesis and reactivity of organometallic gold‐fluoride complexes in oxidation states of Au^(I)^, Au^(II)^, and Au^(III)^, up to and including 2024, are reviewed herein. Despite the flourishing field of gold catalysis, these complexes had long been elusive due to their instability. A widespread interest in C−C and C−F coupling reactions has resulted in several reports of these complexes in recent years. The use of a variety of supporting ligands have facilitated access to these complexes, which has allowed their reactivity to be further studied and understood, thereby laying the ground for future reaction development. This review highlights these advances, organised by the formal oxidation state of the gold centre and the supporting ligand.

## Graphical abstract

In this review, the synthesis and reactivity of organometallic gold‐fluoride complexes in oxidation states of Au^(I)^, Au^(II)^, and Au^(III)^ are summarised. A widespread interest in C−C and C−F coupling reactions has resulted in several developments of these complexes with a range of supporting ligands, which has enabled their reactivity to be studied. Herein, these advances are highlighted, organised by the oxidation state of Au and supporting ligand.

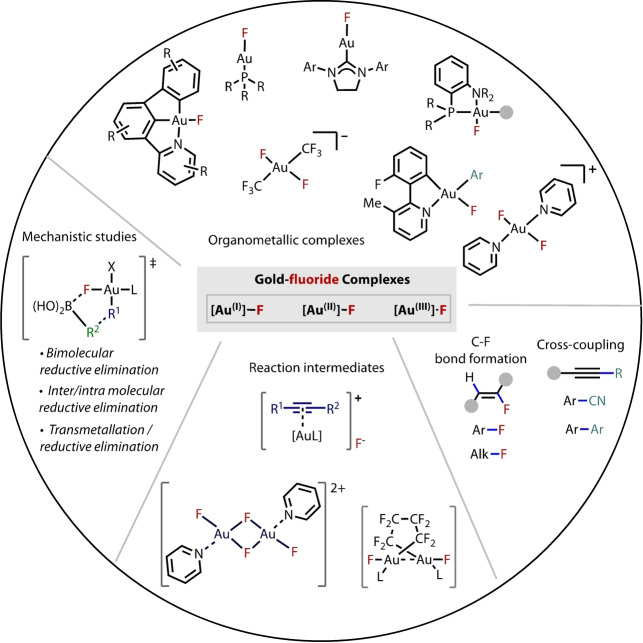

## 1 Introduction

Over the past 3 decades, interest in the development of organic transformations catalysed by gold has increased steadily and considerably. The attraction to gold catalysis is rooted in relativistic effects that are especially prominent in this element.^[1]^ The d and f orbitals of gold are diffuse, while the s and p orbitals are contracted, resulting in low energies of the outer s and p orbitals. These features relate to a lower‐lying LUMO in cationic gold complexes, a high electronegativity (Pauling: 2.54), and highly covalent bonds formed with carbon ligands. Accordingly, the “soft” cationic Au^(I)^ exhibits unique carbophilic Lewis acidity that is far superior to group 10 and other 11 metals, activating alkenes,[^2^, ^3^, ^4^, ^5^, ^6^] alkynes^[7]^ and allenes,^[8]^ towards nucleophilic attack. Au^(III)^ species, despite being “harder” Lewis acids, have also been employed in such catalysis.^[9]^

Considering such burgeoning applications of gold catalysis, together with the importance of organofluorine compounds in medicinal and agro‐chemistry,[^10^, ^11^, ^12^, ^13^] it is no surprise that such catalysts have been employed in the development of C−F bond formations. In this vein, gold‐fluoride complexes have been a source of intrigue. These complexes are rather difficult to prepare, but have been exploited for, and implicated in, an abundance of coupling and C−F bond formation reactions.^[14]^

**Figure 1:**
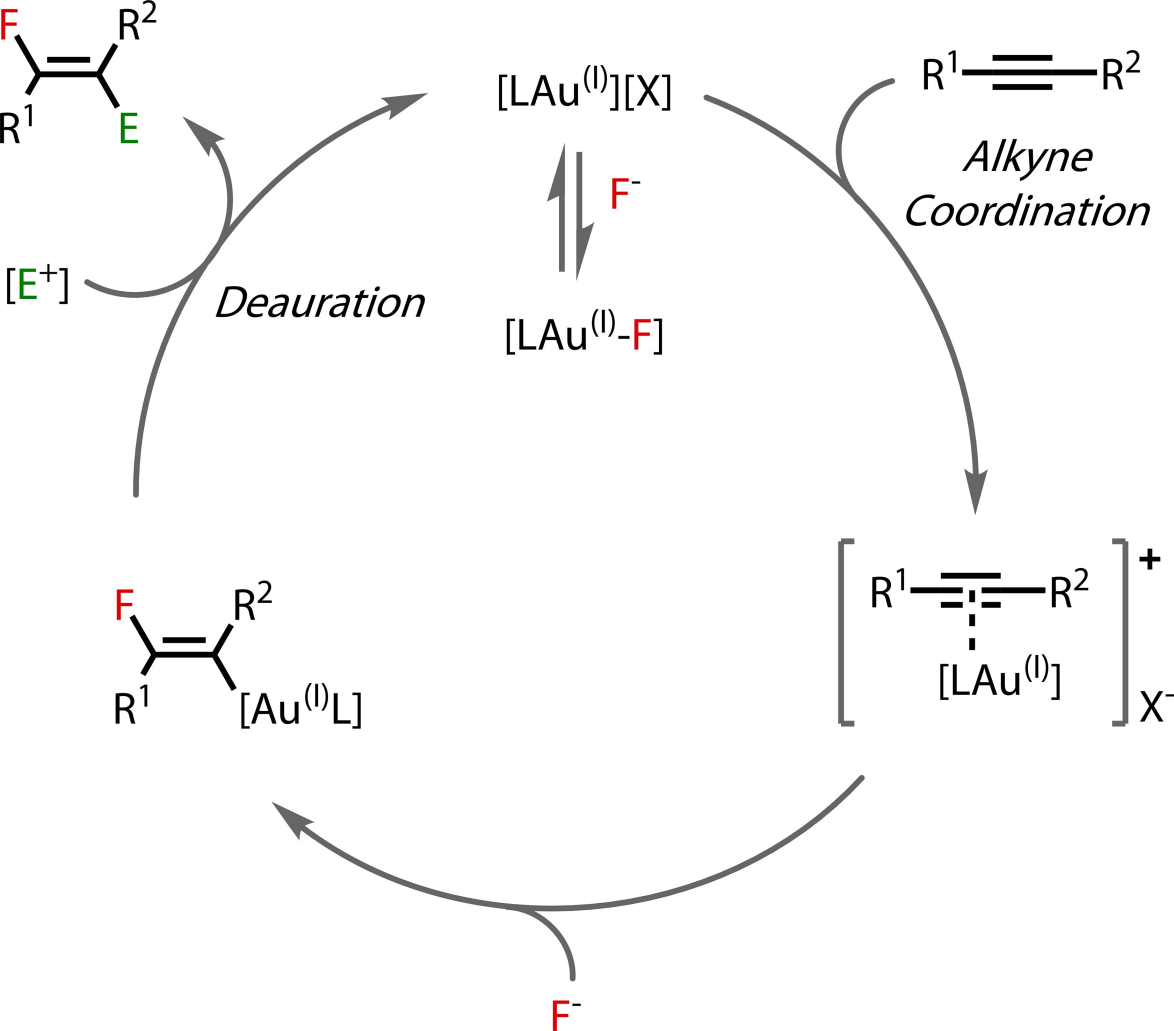
Isohypsic cycle: alkyne fluorination with an electrophile carrying out the deauration step.

The mismatch between the “soft” Au^(I)^ centre and “hard” fluoride ligand, along with the repulsive interaction between the filled metal d orbitals and lone pairs on the fluoride ligand gives rise to a labile Au^(I)^‐F bond, able to add across double and triple bonds in nucleophilic fluorination reactions (Figure 1). The fluoro‐vinyl‐Au^(I)^ species undergoes a deauration step with an electrophile, regenerating the cationic Au^(I)^ species.

Contrastingly, the higher stability of the Au^(III)^‐fluoride bond affords resistance towards C−F reductive elimination. A challenge in this field has been the design of ligands that facilitate this elementary step to enable future developments in oxidative C−F bond formations employing sub‐stoichiometric gold. On the other hand, the reluctance to undergo C−F reductive elimination permits facile transmetalation of the fluoride ligand with boron‐based coupling partners. Several reports of gold‐catalysed oxidative reactions that employ electrophilic fluorine‐based oxidants propose such intermediates,^[14]^ accessed via a putative Au^(I)^ to Au^(III)^ oxidation.

**Figure 2:**
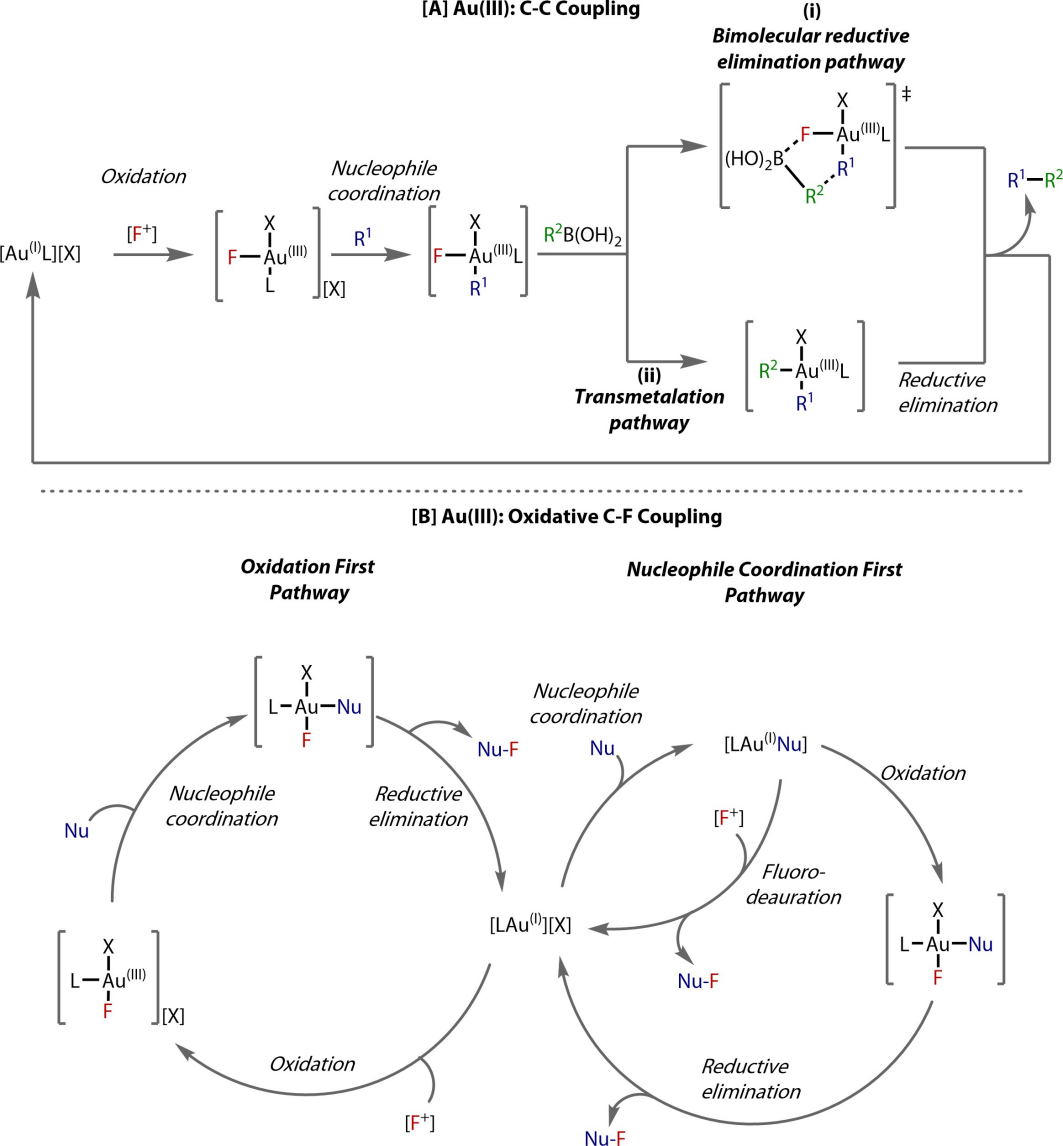
Proposed mechanisms for Au^(I)^/Au^(III)^ cycles involving Au^(III)^‐F intermediates. (A) C−C coupling pathways, undergoing (i) bimolecular reductive elimination, or (ii) sequential transmetalation/reductive elimination. (B) C−F coupling pathways. Left: oxidation of Au^(I)^ prior to nucleophile coordination. Right: Coordination of the nucleophile to the Au^(I)^ centre, with subsequent fluoro‐deauration, or oxidation to Au^(III)^‐F followed by reductive elimination.

Two classes of such reactions can be considered: (1) cross‐coupling (Figure 2A) involving concerted bimolecular reductive elimination (Figure 2A‐(i)), or sequential transmetalation at the fluoride ligand, followed by reductive elimination (Figure 2A‐(ii)), and (2) electrophilic fluorination reactions with C−F bond formation (Figure 2B). Coordination of the nucleophile to Au^(I)^ (nucleophile coordination first pathway, Figure 2B, right), followed by fluoro‐deauration with [F^+^] oxidant, or oxidation by [F^+^] to the putative Au^(III)^‐F intermediate which then undergoes C−F reductive elimination. Alternatively, Au^(I)^ can be oxidised *prior* to nucleophile coordination (oxidation first pathway, Figure 2B‐left), subsequently undergoing the C−F reductive elimination step. Although catalytic Au^(I)^/Au^(III)^ redox cycles posed as a challenge due to the high oxidation potential of Au^(I)^ (1.41 V) relative to Pd^(0)^/Pd^(II)^ (0.92 V),^[15]^ recent years have seen the emergence of multidentate ligand classes that enable facile oxidation of the gold centre,^[16]^ realising powerful cross‐coupling reactivity typically associated with group 10 metals. As such, the importance of Au^(III)^‐F intermediates provides motivation for the study of their synthesis and behaviour for future reaction development.

Since the review on this area by Toste & Wolf in 2014,^[17]^ there have been many exciting developments in this area, with a catalogue of gold fluoride complexes growing significantly in recent years. This review represents a timely report of the published organo Au^(I)^‐, Au^(II)^‐, and Au^(III)^‐fluoride complexes, including those detected as intermediates despite not being isolated, with the aim to discuss their preparation, structural features, and reactivity. Particular attention is devoted to their potential role in gold‐catalysed organic transformations. This review has been organised by the oxidation state of gold and sub‐categorised by supporting ligands.

### 1.1 Early Reports of Gold‐Fluoride Species

The synthesis of binary Au^(I)^‐fluoride, Au^(I)^F, was once considered unachievable,^[18]^ with enthalpy calculations predicting its structure to be less stable than its elemental states (ΔH_f_=+39–45 kcal mol^−1^). The isolation of Au^(I)^F attracted attention because heavier Au^(I)^X (X= Cl, Br, I) had already been described in the literature, but isolation of Au^(I)^F had thus far been unsuccessful. Despite many theoretical and experimental studies, only ambiguous spectroscopic data had been observed, such as the 1992 work by Saenger & Sun,^[19]^ who observed an emission band for Au^(I)^F that was inconclusive.

It was not until 1994 when definitive evidence for neutral Au^(I)^F was reported by Schwarz, who used neutralisation‐reionisation mass spectrometry.^[20]^ Since then, there have been numerous more successes, including from Evans & Gerry,^[21]^ who used micro‐spectroscopy to measure the length of the Au^(I)^F bond to be 1.918 A. The bond strength of Au^(I)^F was calculated to be 68–85 kcal mol^−1^. Interestingly, this was within the range for heavier halide bond strengths, Au‐X, where X=Cl, Br, I at 78, 68, 66 kcal mol^−1^, respectively.

## 2 Au^(I)^‐Fluoride Complexes

### 2.1 NHC Ligands

After facing the difficulties in accessing homoleptic Au^(I)^‐fluoride, focus turned to synthesising stable ligand‐supported Au‐fluoride complexes. Early reports of these complexes employed N‐heterocyclic carbenes (NHCs) as ligands. NHCs are ancillary ligands that can bind to both low and high oxidation state metals^[22]^ via a single M−C σ‐donating interaction,^[23]^ and can stabilise Au‐F bonds by withdrawing electron density from the filled dπ‐pπ anti‐bonding Au‐F orbitals.^[24]^ Unlike monodentate phosphines, NHCs are resistant towards ligand oxidation and, in turn, can prevent the reduction of either Au^(I)^ or Au^(III)^ to Au^(0)^. Furthermore, NHC ligands are typically more sterically demanding than phosphines, which prevents Au^(I)^‐ aggregation. The insufficient stabilising effects of phosphine were illustrated in an early unsuccessful attempt to synthesise [(PPh_3_)AuF]) through direct Cl/F exchange of [(PPh_3_)AuCl], reported by Schmidbaur in 2000.^[25]^ Instead, the {[(Ph_3_P)Au]_3_O}[H_2_F_3_] complex formed, presumably via the desired [(PPh_3_)AuF], followed by rapid hydrolysis, with such behaviour differing greatly to the corresponding chloride and bromide complexes. These observations demonstrated the poor affinity between Au^(I)^ and fluoride, in addition to a need for alternative ligand classes to access these complexes.

**Figure 3:**
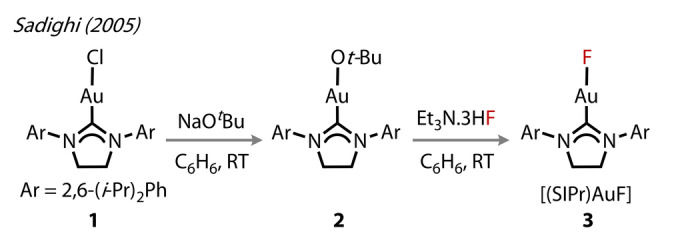
Sadighi's (2005) route to [(SIPr)AuF] **3**.

The first Au^(I)^‐F complex, [(SIPr)AuF], **3** (SIPr=1,3‐bis(2,6‐diisopropylphenyl)imidazolin‐2‐ylidene, was reported by Sadighi and co‐workers in 2005 (Figure 3).^[24]^ Owing to the increased lability of the Au‐O*t*‐Bu bond, compared to Au‐Cl, the complex was synthesised by initially preparing precursor **2** by reacting NaO*t*‐Bu with **1** in benzene. The labile Au‐O*t*‐Bu bond enabled facile substitution by fluoride using NEt_3_ ⋅ 3HF to yield **3**. Crystals of **3** were grown in DCM, revealing a monomeric linear complex, and with a Au^(I)^‐F bond length of 2.028 Å, which is reportedly slightly longer than that measured for Au^(I)^‐F (1.918 Å).^[21]^ Complex **3** was stable in the solid state, showing no decomposition when protected by light, but underwent ca. 25 % decomposition in solution (DCM) over one day.

The Au^(I)^‐F interactions of **3** were analysed by DFT calculations, which showed significant contribution of fluoride p‐orbitals to the HOMO‐1, and HOMO‐2 (around 60% fluoride contribution), exhibiting dπ‐pπ Au‐F antibonding combinations, which contrasts with d_8_ iridium‐F complexes.^[26]^

**Figure 4:**
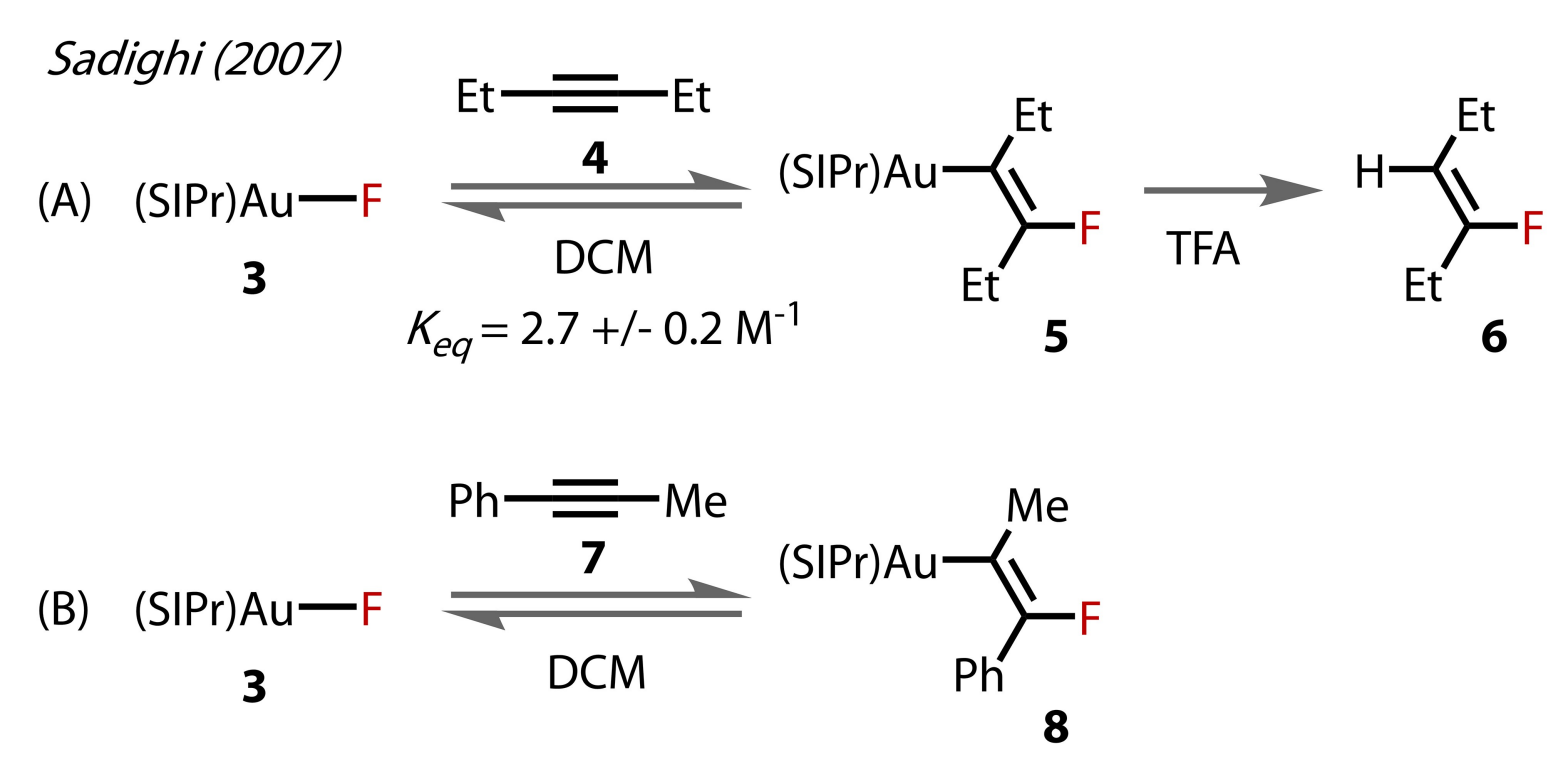
Alkyne fluorination mediated by [(SIPr)AuF] **3**.

In a 2007 study by the same group, [(SIPr)AuF] **3** displayed reactivity towards 3‐hexyne **4** by reversible insertion into the Au^(I)^‐F bond,^[27]^ yielding β‐(fluorovinyl)Au complex, **5** (Figure 4A). Protodeauration of **5** with trifluoroacetic acid (TFA) afforded the corresponding hydrofluorination product, **6** (Figure 4B). The same Au^(I)^‐F insertion process was observed with 1‐phenyl‐1‐propyne **7** and upon its crystallisation, the *trans*‐arrangement of the β‐(fluorovinyl)Au **8** was confirmed by x‐ray diffraction. When dissolved in DCM, the equilibrium was re‐established, showing **8** to be unstable in solution.

**Figure 5:**
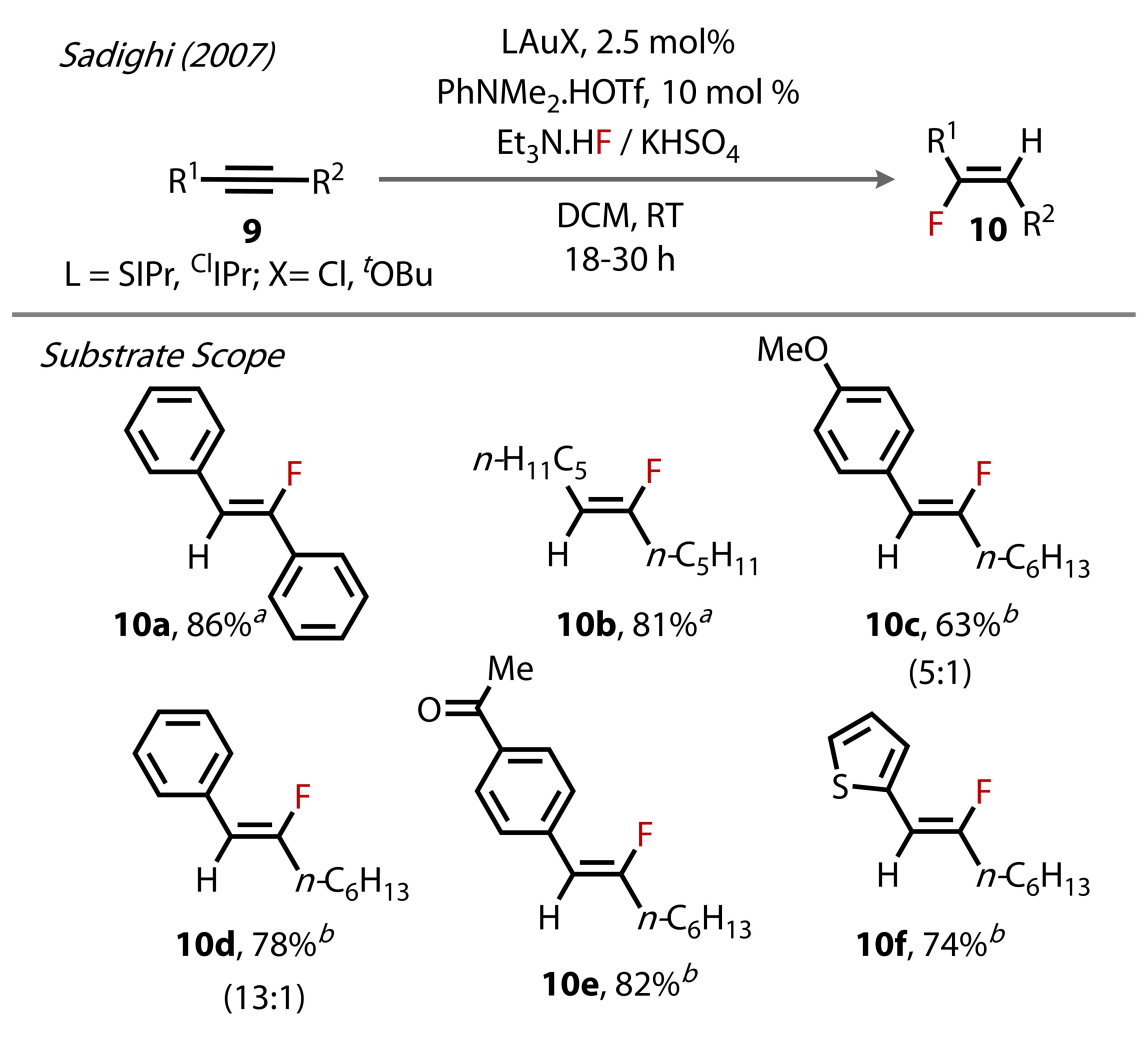
Sadighi's (2007) hydrofluorination of alkynes, with regioisomer ratios where appropriate (the structure shown is the major regioisomer). [a] (ClIPr)AuCl/AgBF_4_ pre‐catalyst. [b] [(SIPr)Au(O*t*‐Bu)] **2** precatalyst.

The authors proceeded to develop a catalytic protocol for the hydrofluorination of internal alkynes, providing a direct method to fluoroalkenes from alkynes at room temperature (Figure 5). Various internal alkynes **9** were investigated, with the authors observing exclusive *trans*‐hydrofluorination for all substrates. Symmetrical conjugated (**10** **a**) and non‐conjugated (**10** **b**) alkynes were high yielding. For non‐symmetrical alkynes, a slight trend was observed, where more electron‐deficient substrates gave rise to higher regioselectivities (**10** **e** vs **10** **c** and **10** **d**). Bulkier supporting NHCs, such as SIPr and ClIPr, gave rise to higher yields, with less sterically demanding NHC or phosphine ligands resulting in the precipitation of Au^(0)^ and poor conversion. Since this report, multiple variations of this method have been explored for both internal and terminal alkynes.[^28^, ^29^, ^30^, ^31^, ^32^, ^33^, ^34^, ^35^, ^36^, ^37^]

**Figure 6:**
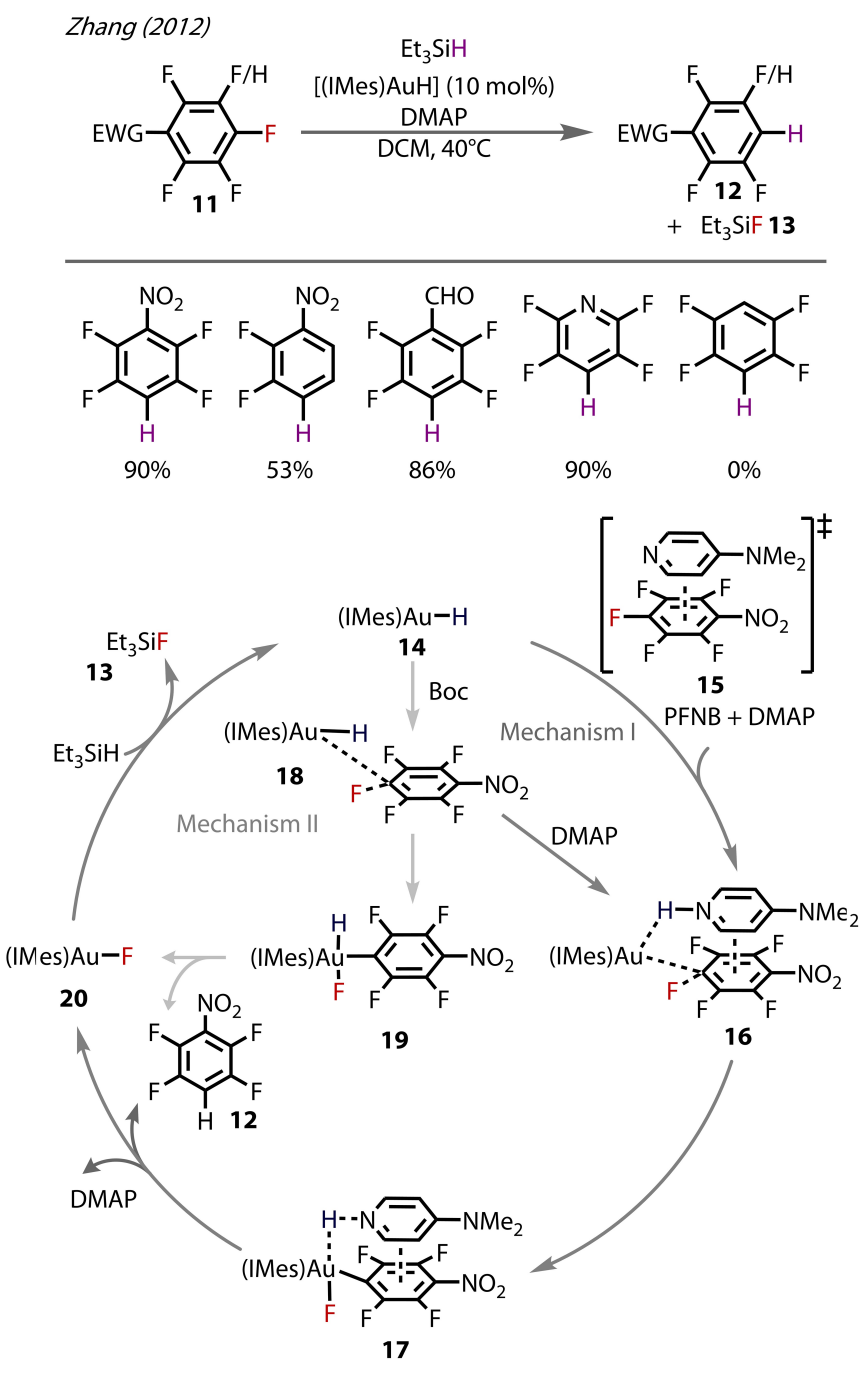
Proposed mechanism for Zhang's hydrodefluorination of fluoroarenes catalysed by [(IMes)AuH] **14**. DMAP=4‐Dimethylamino pyridine.

In 2012 Zhang provided evidence of a Au^(I)^‐fluoride catalytic intermediate in the hydrodefluorination of fluoroarenes **11** (forming **12**) using Au^(I)^‐H complex **14** (Figure 6).^[38]^ Based on experimental and theoretical observations, the mechanism was proposed to proceed through two routes, depending on the presence (mechanism I) or absence of DMAP (mechanism II). With DMAP present (mechanism I), the arene substrate and DMAP engage in a weak π‐π interaction (**15**), before forming the protonated DMAP species **16**, followed by oxidative addition to form **17**. From this stage, reductive elimination of **17** forms the hydrodefluorination product, along with the Au^(I)^‐fluoride intermediate, [(IMes)AuF], **20** IMes=N,N′‐bis(2,4,6‐ trimethylphenyl)imidazolin‐2‐ylidene). In the absence of DMAP (mechanism II), [(IMes)AuH] **14** initially interacts with the π‐system of the arene substrate (**18**), then forming **19** by oxidative addition, as supported by DFT calculations. [(IMes)AuF] **20** was characterised by ^19^F NMR, which showed a signal at −248.61 ppm that matched the shift of other NHC‐supported Au^(I)^‐fluorides. The presence of Au^(I)^‐fluoride in the catalytic cycle was further validated by adding HSiEt_3_ to a solution of **19**, from which F‐SiEt_3_ was observed by ^19^F NMR. The same group also demonstrated perfluoroarene hydrodefluorination using Xantphos/*t*‐BuXantphos ligands,^[39]^ though did not provide experimental evidence for Au‐F species, and instead proposed such intermediates through DFT calculations.

**Figure 7:**
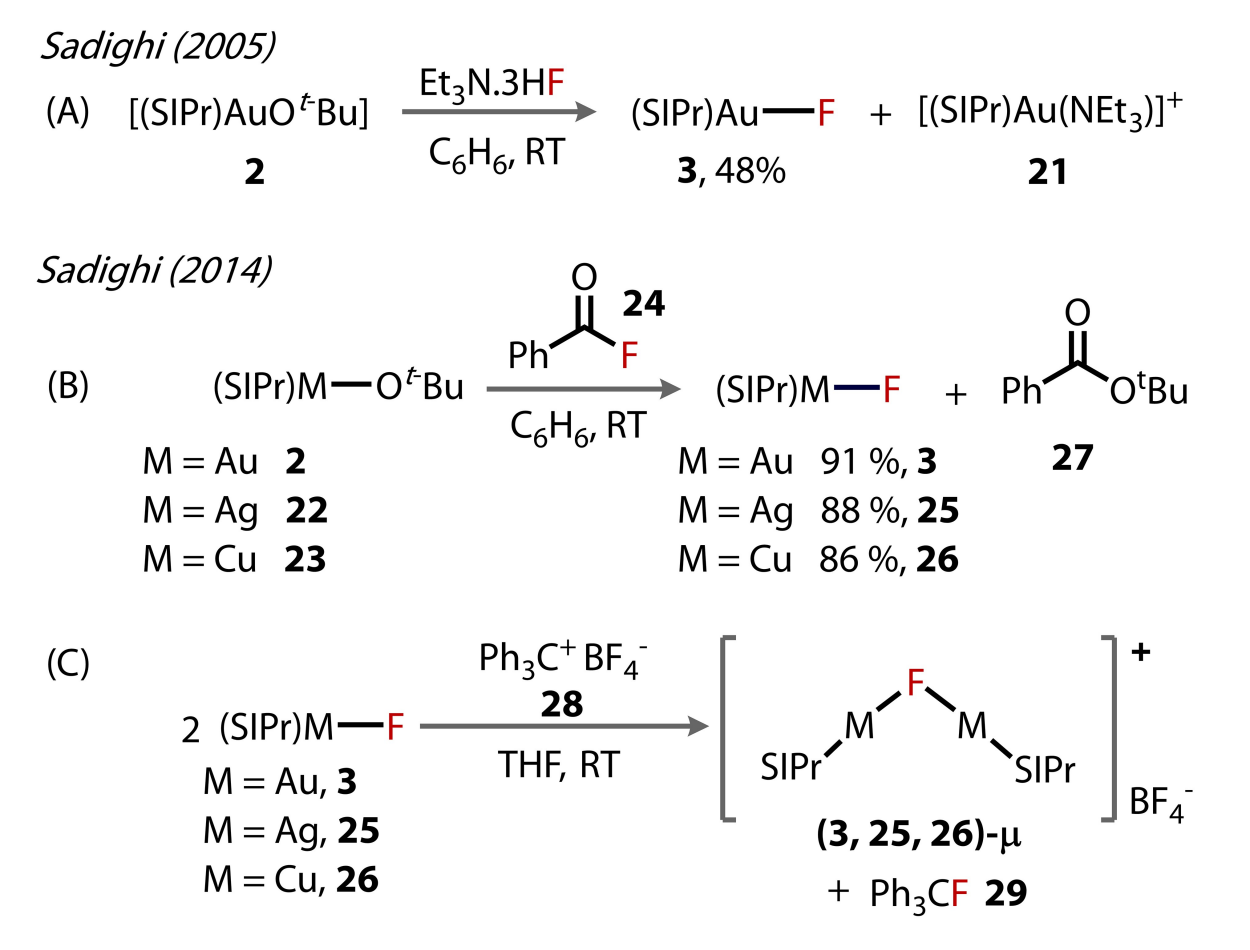
Routes to [(SIPr)MF] **3**, **25**, and **26**, from [(SIPr)MOtBu] by (A) original method by Sadighi in 2005 and (B) improved method by Sadighi (2014). (C) Formation of bridging fluoride complexes **3‐μ**, **25‐μ**, and **26‐μ** by fluoride abstraction with Ph_3_C^+^BF_4_^−^ **28**.

Dinuclear Au^(I)^‐fluorides were first reported in 2014 by Sadighi, who synthesised a series of group‐11 transition metal μ^2^‐fluoride‐bridging dinuclear cationic complexes from the terminal fluoride complexes, [(SIPr)MF] (M=Cu, Ag and Au) (Figure 7).^[40]^ The original 2005 synthesis of terminal Au^(I)^‐fluoride complex **3** exhibited reproducibility issues due to the hygroscopic nature of the 3HF ⋅ NEt_3_ reagent, and the observation of [(SIDipp)Au(NEt_3_)]^+^ **21** (Figure 7A).^[24]^ A higher‐yielding procedure was pursued involving the addition of benzoyl fluoride **24** to a solution of [(SIPr)Au(O*t*‐Bu)] **2**, which cleanly produced the desired complexes, [(SIPr)AuF] **3** (with the corresponding copper and silver complexes also formed using this protocol, **25** and **26** from **22** and **23**) (Figure 7B), while the *t*‐butyl benzoate by‐product **27** does not affect the course of the reaction. [(SIPr)Au]_2_(μ‐F)}[BF_4_] **3‐μ** (in addition to the copper and silver complexes **25‐μ** and **26‐μ**) was subsequently synthesised by abstraction of the fluoride from **3** using 0.5 equivalents of triphenylcarbenium tetrafluoroborate **28**, forming alongside the (fluoromethanetriyl)tribenzene by‐product **29** (Figure 7C).

In line with the lability of the Au^(I)^‐F bond, **3‐μ** required the most rigorously dry conditions compared to **25‐μ** and **26‐μ**, with **3‐μ** being prone to hydrolysis to form the corresponding hydroxy‐bridged species. The dinuclear structures showed no further aggregation, a result of the steric demand imposed by the NHC ligands. The Au^(I)^‐F bonds were slightly longer in the bridged complex compared to the terminal (2.060 Å versus 2.028 Å).^[24]^

**Figure 8:**
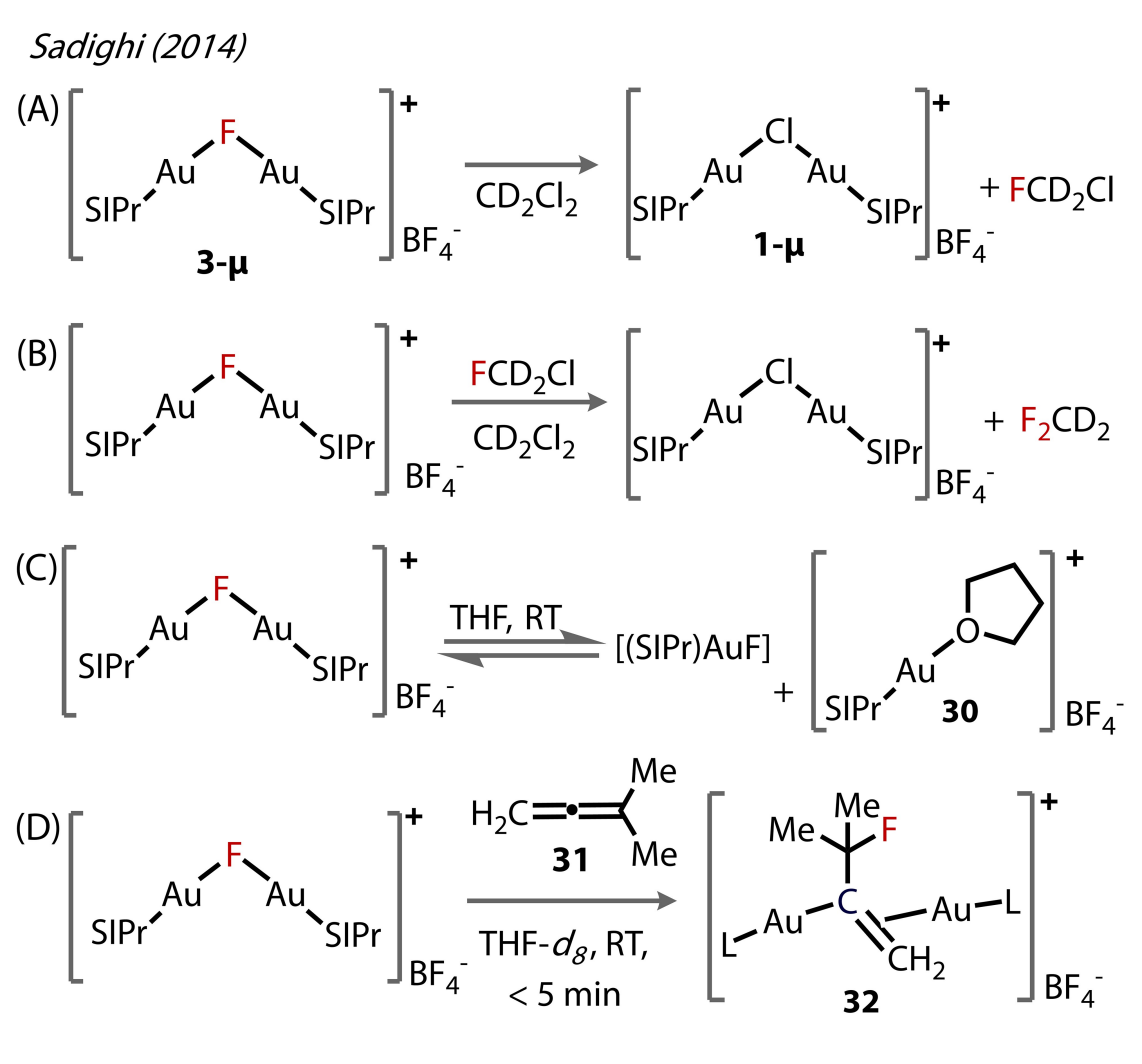
(A) Activation of the C−Cl bond of [{(SIPr)Au)}2(μ‐F)_2_]+[BF_4_]^−^ **3‐μ** in CH_2_Cl_2_ and (B) FCD_2_Cl formation through Cl/F exchange by **3‐μ**. (C) Reversible coordination of THF to **3‐μ** to form THF‐adduct **30**. (D) **3‐μ** engaging in Au‐F addition across an allene C=C bond forming the di‐aurated species **32**.

Unlike the corresponding silver and copper complexes **25‐μ** and **26‐μ**, which showed minimal decomposition in DCM‐*d* ^*2*^ after a day, **3‐μ** was completely consumed within 24 hours at room temperature, due to chloride abstraction from DCM‐*d* ^*2*^ (Figure 8a and b). The activation of C−Cl bonds was presumably due to both the higher affinity of Au for chloride than fluoride compared to the other metals and the high lability of the Au^(I)^‐F bond. The authors propose the displacement of the fluoride ligand by THF forming complex **30** (Figure 8C), resulting in rapid interconversion of the bridged fluoride complex with the terminal Au‐F and solvent‐coordinated complexes.

The reactivity of the bridged complexes towards allenes was examined, using 3‐methyl‐1,2‐butadiene **31** in THF‐*d*_8_. The reaction was rapid, with ^1^H NMR showing complete consumption of **3** within 5 minutes at room temperature to form **32**. By contrast, the silver complex **25‐μ** only gave rise to minimal product, and no reaction was observed for the copper complex **26‐μ**. The authors proposed a diaurated species **32**. Similar diaurate species are relevant in various Au^(I)^‐catalysed reactions involving allenes,^[41]^ alkynes,[^42^, ^43^, ^44^, ^45^] and arenes.[^46^, ^47^, ^48^] To the best of our knowledge, however, gold‐catalysed nucleophilic fluorination of allene substrates has yet to be explored.

### 2.2 Phosphine Ligands

**Figure 9:**
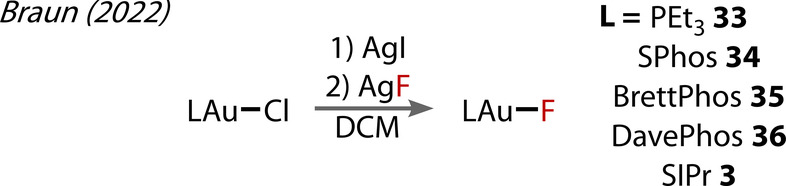
Braun‘s (2020) preparation of phosphine Au^(I)^‐F complexes **3**, and **33**–**36**.

Braun (2022) prepared (L)Au^(I)^‐F complexes with a series of phosphine ligands (SPhos, DavePhos, BrettPhos) through sonication of the iodide complex with AgF (Figure 9), proceeding with quantitative conversion within minutes in most cases.^[49]^ While the use of PPh_3_, and PPh_2_py (2‐(diphenylphosphanyl)pyridine) as ligands proved unsuccessful, the use of PEt_3_ yielded an unidentifiable side product alongside the desired complex, which was attributed to the poor electron‐donating ability and reduced steric demand of these ligands. Of note, these Au^(I)^‐fluoride complexes (**33**, **34**, **35**, **35**, **3**) could not be accessed by directly reacting the Au^(I)^‐Cl complexes with AgF.

The calculated structural and bonding properties were all within a small range (Table 1), showing no clear trend with respect to the ligand. To gain insight into the reactivity of these complexes, the free energy change for the Cl/F substitution through HCl/HF was also calculated, showing a substantial thermodynamic preference for the chloride complex formation (ranging from −21.5 to −22.2 kcal / mol across these complexes). This calculated free energy change was barely affected by the ligand *trans* to the fluoride/chloride. The major difference was the Au‐F ^19^F NMR shift in the SIPR complex **3**. This effect was attributed to the paramagnetic shielding contribution, σ_p_, present in the SIPr complex.

**Table 1:** Structural features of various phosphine Au^(I)^‐F complexes **3** and **34**–**36** from Braun's (2022) study.

[Au(F)(L)]	δ(^19^F)	*q*(F)^[A]^	*q*(Au)^[a]^	*r*(Au‐F)^[b]^
(SPhos) **34**	−204.3	−0.75	0.45	1.972
(BrettPhos) **35**	−208.1	−0.72	0.47	1.947
(DavePhos) **36**	−204.6	−0.75	0.46	1.974
(SIPr) **3**	−259.9	−0.73	0.51	1.954

[a] q=natural atomic charges in au, [b] Au‐F bond lengths (Å).

**Figure 10:**
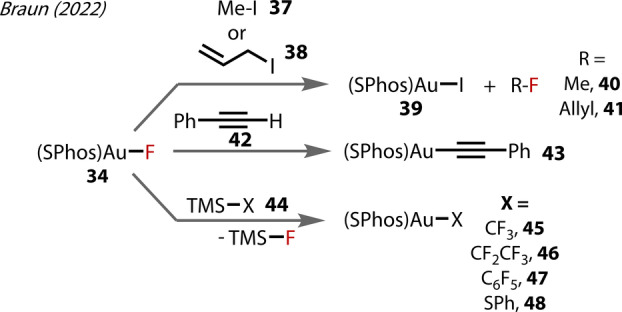
(SPhos)Au‐F **34** reactivity reported by Braun (2022).

Au^(I)^ species **34** mediated several reactions (Figure 10). Methyl‐ and allyl‐iodide (**37**, and **38**) underwent fluoride substitution (**40** and **41**), yielding as the by‐product the Au‐I complex **39**. The familiar Au‐alkynyl complex readily formed upon reaction with terminal and TMS‐phenyl acetylene **42**, forming **43**. Transmetallation was also facile with various TMS‐X reagents **44** (forming **45**–**48**).

**Figure 11:**
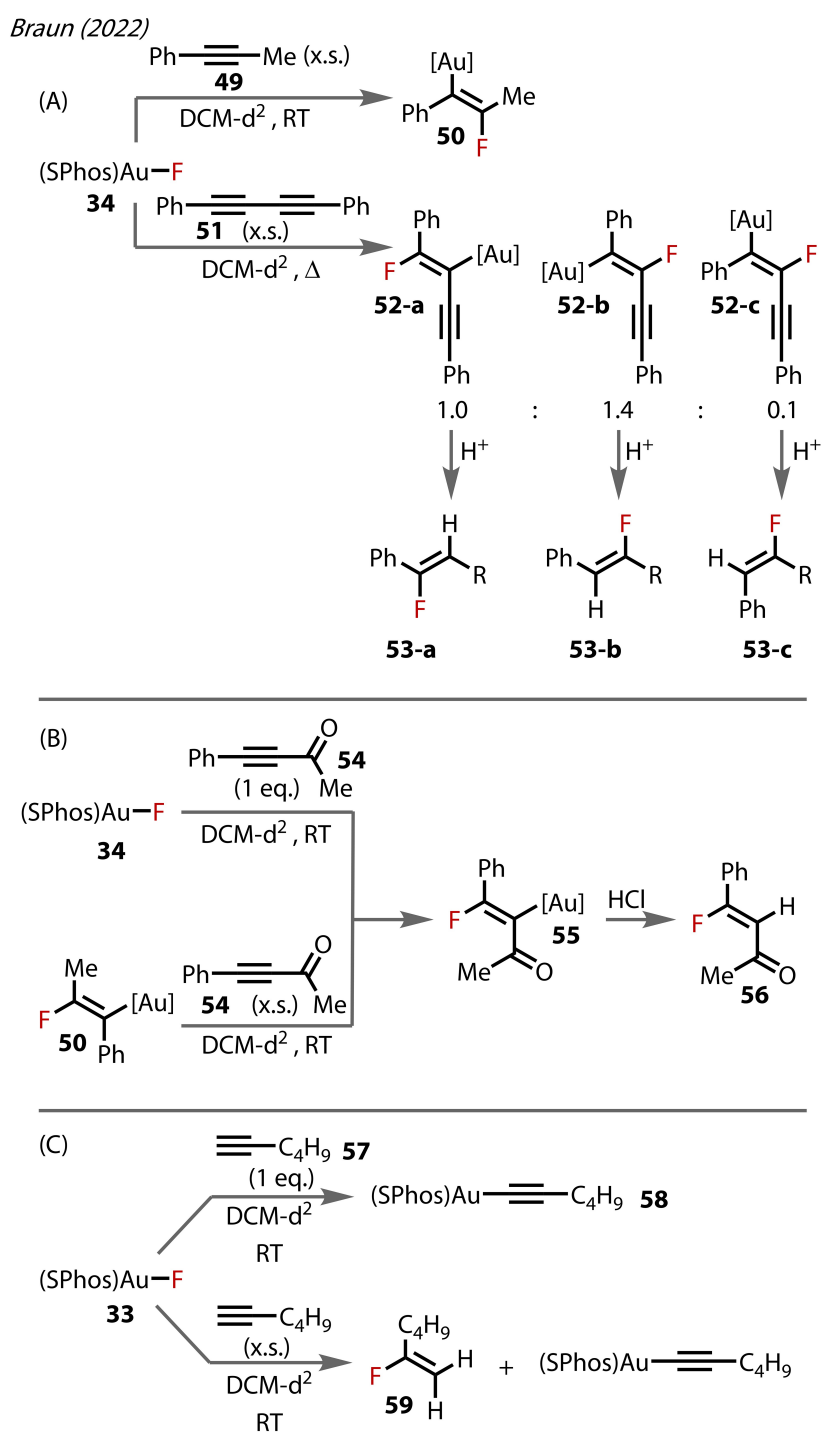
Braun's (2022) study on [(SPhos)Au‐F] **34** reactivity with various alkynes.

In a separate 2022 report, Braun studied [(SPhos)Au^(I)^‐F] **34** towards alkyne hydrofluorination Figure 11).^[50]^ The same regioiselectivity (forming **50**) was observed for Au‐F/alkyne insertion as in Sadighi's 2007 study with the SIPr complex **3**.^[27]^ Interestingly, the internal diyne **51** ‐ a substrate class not explored in Sadighi's study ‐ underwent the same insertion to **52** (Figure 11A). Proto‐deauration occurred (forming **53**) without further reaction of the fluorinated ene‐yne, with this result being consistent across various forms of the cationic complex, i.e. different counter‐anions to the cationic SPhos‐Au^(I)^. The insertion process gave not only an expected mixture of regioisomers (**52‐a** : **52‐b**, 1.0 : 1.4), but also a small amount of the formal *syn*‐Au‐F insertion **52‐c**. Importantly, this *syn*‐Au^(I)^‐F insertion pathway has only been scarcely reported prior, with limited examples of the corresponding hydrofluorination product from directing group‐tethered alkynes.^[32]^ Without a directing group, this *syn‐*Au‐F pathway may be more feasible with diyne substrates. The electron‐deficient alkyne **54** also formed the corresponding fluoro‐vinyl complex **55**, which proto‐deaurated with HCl to yield the corresponding *Z*‐hydrofluoroalkene **56**, (Figure 11B). Subjecting the vinyl complex **50** to an excess of alkyne **54** resulted also in complex **55**, presumably by first reverting to the Au^(I)^‐F. In contrast to Sadighi's study,^[27]^ a terminal alkyne substrate **57** was examined in this insertion process with Au^(I)^‐F. Insertion was not directly observed (Figure 11C), rather the Au^(I)^‐alkynyl species **58** was formed in the presence of 1 equivalent of the alkyne. However, the hydrofluorination product was formed when employing excess alkyne. Terminal alkynes may undergo hydrofluorination/proto‐deauration via the Au‐alkynyl species, in this case forming **59**.

**Figure 12:**
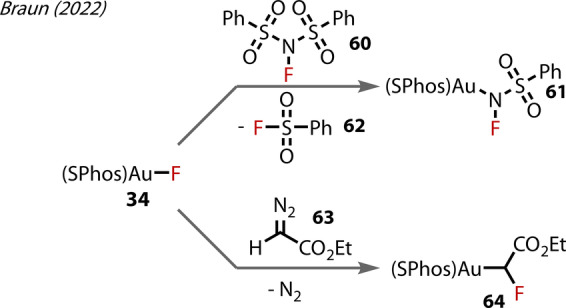
Braun's (2022) formation of fluoroamido‐(SPhos)Au^(I)^ **61** and 1,1‐Au‐F insertion with ethyl diazoacetate to form **64**.

In a separate report in 2022, the same group reported the preparation of a (SPhos)Au^(I)^‐fluoroamido complex **61** (Figure 12),^[51]^ the first example of such a species, via the reaction of the (SPhos)Au‐F **34** and NFSI **60**, with concomitant FSO_2_Ph formation **62**. Finally, a rare example of 1,1‐Au‐F insertion was demonstrated with ethyl diazoacetate **63**, accessing the α‐fluoroalkyl‐Au^(I)^ complex **64**.

**Figure 13:**
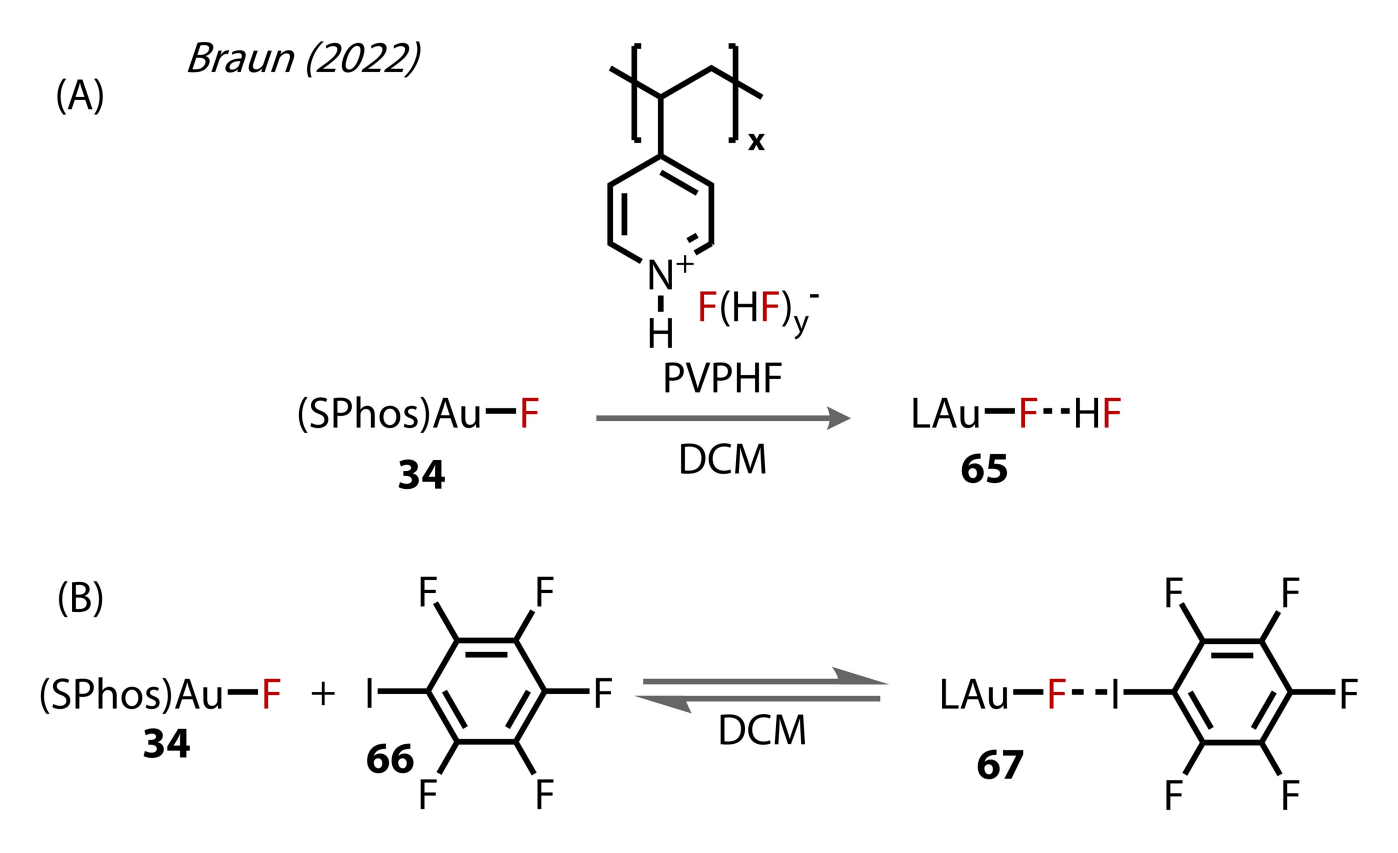
Braun's (2022) study of non‐covalent interactions with (SPhos)Au‐F **34** with (A) hydrogen‐bonded HF (SPhos)Au‐F **65** and (B) halogen bonding with iodopentafluorobenzene (complex **67**).

Braun (2022) examined the H‐bonding and halogen bonding interactions undertaken by (SPhos)Au‐F **34** (Figure 13).^[52]^ The H‐bonded complex **65** was formed by reacting with PVP ⋅ HF (PVP ⋅ HF=poly[4‐vinylpyridiniumpoly(hydrogen fluoride)]) (Figure 13A). The authors provided spectral and computational evidence for the Au‐F ⋅ HF species **65** rather than a symmetrical bifluoride counter‐anion. The ^19^F NMR coupling patterns and integrations are in line with only one HF coordinating to the fluoride ligand. DFT‐optimised structures calculated the F−F distance in this complex to be 2.388 Å, differing notably from that calculated for the [FHF]^−^ *r*_F⋅⋅F_=2.269 Å, while the H−F bond length (0.979 Å) is close to the non‐H‐bonded distance (0.925 Å). Finally, these data show the proton residing in an unsymmetrical position between both fluorides (*r*_(Au)F⋅⋅H(F)_=1.409 Å, *r*_(AuF⋅⋅)H−F_=0.979 Å). The same H‐bonding Scheme is present when including up to two DCM molecules explicitly (as hybrid explicit/implicit solvation). The calculated Au‐F bond expectedly increases slightly from 2.008 to 2.055 Å upon introduction of the H‐bonding interaction. Subjecting the (NHC)Au‐F complexes to the same analysis corroborates a similar hydrogen bonding interaction to the phosphine complex, though this interaction was calculated to be slightly less exergonic (by 6 kJ mol^−1^), with disfavoured interaction with a second HF, either directly at the Au‐F fluoride, or as part of an H‐bonding network.

The halogen‐bonding interactions exhibited by **34** were also explored, using the SPhosAu‐F ⋅ IC_6_F_5_ adduct **67** as an example (Figure 13B). ^19^F NMR titration experiments of **34** and **66** in DCM revealed a substantial change in the chemical shift of the Au‐F environment, moving more downfield with increasing iodoarene concentration relative to the gold complex (−210 ppm to −185 ppm, from without aryl‐iodide **66** to [Ar‐I]/[Au‐F] of ~33). Secondly, a Job plot for this equilibrium determined a 1 : 1 complex in DCM, though with a possibility to form a 2 : 1 Ar‐I : Au‐F complex in the solid state, as determined by their obtained crystal structure of this adduct.

**Figure 14:**
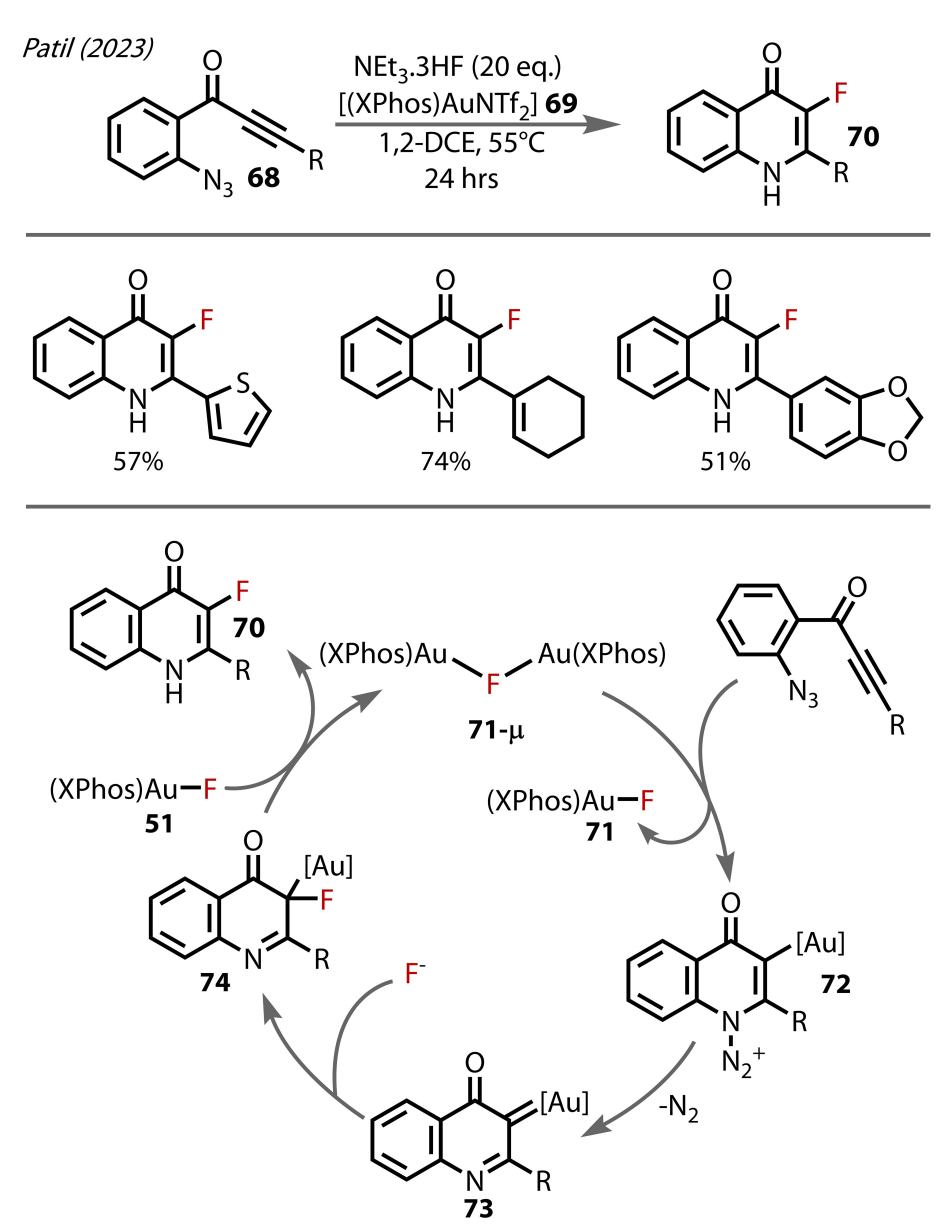
Patil's (2023) formation of C3‐fluorinated aza‐heterocycles involving [(Xphos)AuF] terminal **71** and bridging **71‐μ** complexes formed in situ.

In 2023 Patil reported on the development of a gold‐catalysed method for synthesising C3‐fluorinated aza‐heterocycles **70** from azido‐alkynes **68** via α‐imino Au‐carbene formation (Figure 14).^[53]^ A μ^2^‐fluoride bridging [(XPhosAu)_2_F].NTf_2_ complex **71‐μ** was isolated after treating [(XPhos)AuNTf_2_] **69** with NEt_3_ ⋅ 3HF, which was the first example of a phosphine‐supported fluoride‐bridged Au^(I)^ complex. There may be a stabilising interaction in **71‐μ** between the π‐system of the XPhos ligand and its nearby gold centre, and the Au‐Au distance of 3.468 Å is indicative of weak aurophilic interactions. **71‐μ** was proposed to be responsible for the activation of the alkyne substrate in this transformation rather than the mononuclear Au‐F complex **69**, a proposal that was based on their control experiments, corresponding to the greater ability for π‐activation of the bridging complex. The azide substituent cyclizes onto the Au‐activated alkyne to form species **72**. Nitrogen is released while forming the alpha‐imino Au^(I)^‐carbene species **73**, which undergoes fluorination and proto‐deauration to form the fluorinated aza‐heterocycle **70**.

### 2.3 Trifluoromethyl Complexes

**Figure 15:**
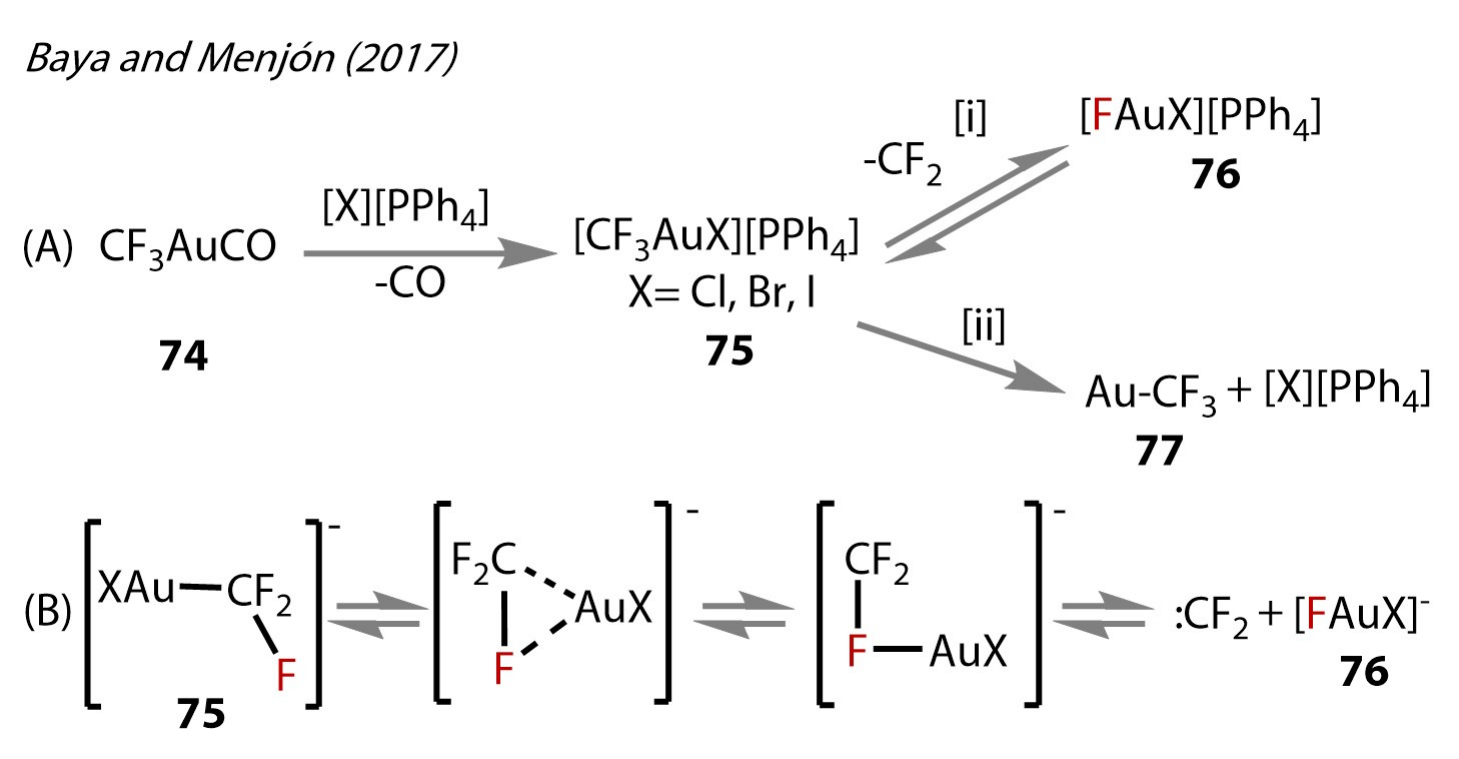
Baya and Menjón's (2017). (A) Synthesis of halide Au‐CF_3_ complexes **75**. (B) CF_2_ carbene dissociation pathway mechanism from **75**.

Given the widespread applications of trifluoromethyl‐containing compounds,^[54]^ and the many reports of Au‐trifluoromethyl complexes,[^55^, ^56^, ^57^, ^58^, ^59^, ^60^, ^61^, ^62^, ^63^] trifluoromethyl ligated Au^(I)^‐fluoride species are surprisingly scarce in the literature. The detection of a series of gaseous fluorohalide‐Au^(I)^ complexes were reported by Baya, Menjón (2017) and co‐workers (Figure 15).^[64]^ Gaseous trifluoromethyl Au^(I)^‐derivatives of the form [PPh_4_][CF_3_AuX] (X=Cl, Br, I) (**75**) were prepared by exchanging the labile CO ligand of [CF_3_AuCO] (**74**) with halide (Figure 15A[i]). Collision‐induced activation resulted in dissociation of free CF_2_ from the complex, and the detection of Au^(I)^‐fluoride species [FAuX]^−^ (X=Cl, Br, I) **76** in the gas‐phase by tandem mass‐spectroscopy. Results showed competitive ligand dissociation reactions under these conditions forming Au‐CF_3_ **77** (Figure 15A[ii]). DFT calculations (M06) predicted bond lengths of 2.013–2.029 Å barely changing with respect to the *trans‐*halide ligand.

The mechanism for decomposition of **75** to the corresponding [XAuF]‐(X=Cl, Br, I) **76** was proposed to proceed via Au^(I)^‐C bond weakening, followed by strengthening of the Au^(I)^‐F interaction. The CF_3_ ligand engages in μ_2_‐coordination with the Au^(I)^ centre (Figure 15B). Complete cleavage of the Au^(I)^‐C bond gives rise to a fluoride‐bridged F_2_C‐F‐Au^(I)^ species with a calculated Au^(I)^‐F bond length of 2.15 Å (compared to ~2.06 Å in Sadighi's [{(SIPr)Au}_2_(μ‐F)] **3‐μ**. The CF_2_ carbene then dissociates, releasing the [XAuF]^−^ anion **76**. Their calculations suggest the reverse reaction—difluorocarbene insertion into the Au^(I)^‐F bond—to be essentially barrierless.

With thorough fundamental structural and reactivity studies on various Au(I)‐F complexes, along with the multiple reports of gold‐catalysed alkyne hydrofluorination, many future research directions should be pursued. Systematic studies on the effects of ligand electronic and steric properties on the rate, regio‐, and stereo‐selectivity of Au‐F insertion reactions would be of value. For example, (SPhos)Au‐F **34** requires more forcing conditions to achieve the Au‐F alkyne insertion reaction compared to the NHC complex (SIPr)Au‐F **3**. Mechanistic insights into the unexplored *syn*‐Au‐F insertion pathway would also be particularly insightful.

## 3 Au^(II)^‐Fluoride Complexes

Au^(II)^‐fluoride complexes are challenging to study, since gold is most stable in oxidation states 0, 1, and 3.^[65]^ Au^(II)^ is less common, having an unpaired electron in the high energy d_*x*2‐*y*2_ orbital in a square planar geometry, and is readily oxidised to Au^(III)^.^[66]^ Au^(II)^ complexes are more commonly found in dinuclear form and typically engage in Au‐Au (aurophilic) interactions.[^65^, ^67^] With the potential to facilitate redox catalysis to deliver C−F bonds through Au^(II)^‐Au^(II)^ aurophilic interactions (see below),[^67^, ^68^] dinuclear Au^(II)^‐fluoride complexes represent species worthy of study. The isolation of these complexes has proven difficult, and literature examples are elusive.^[69]^

**Figure 16:**
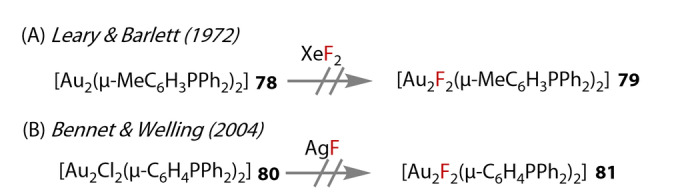
Early unsuccessful attempts at preparing Au^(II)^‐fluoride complexes.

Attempts include those by Leary & Barlett (1972) (Figure 16),^[70]^ who were unsuccessful in inducing oxidative addition of [Au_2_(μ‐2‐MeC_6_H_3_PPh_2_)_2_] **78** with XeF_2_ with the aim of forming [Au_2_F_2_(μ‐2‐MeC_6_H_3_PPh_2_)_2_] **79**. Bennett & Welling^[71]^ also attempted to form [Au_2_F_2_(μ‐C_6_H_4_PPh_2_)_2_] **81** by treating [Au_2_Cl_2_(μ‐C_6_H_4_PPh_2_)_2_] **80** with AgF, but the desired product could not be characterised.

**Figure 17:**
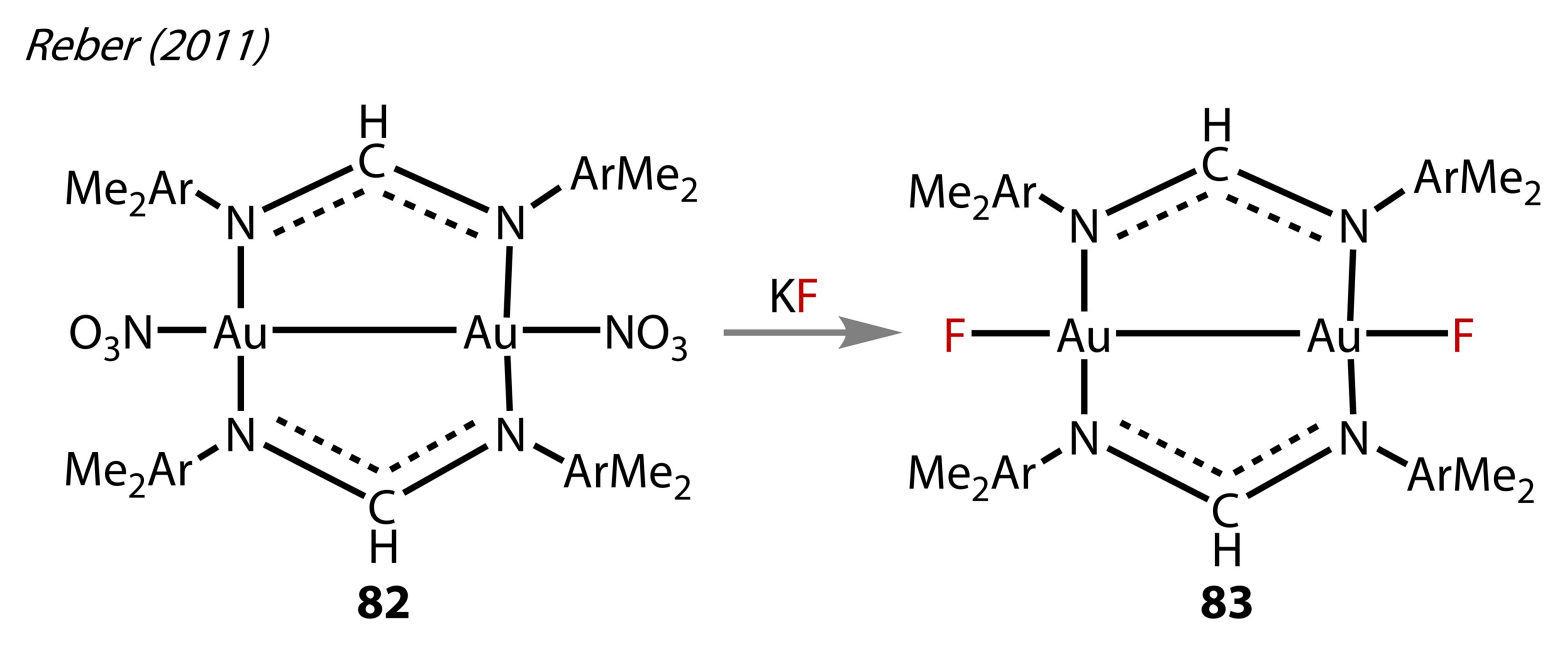
Synthesis of [Au_2_(bis‐(2,6‐dimethylphenyl) formamidinate)_2_F_2_] **83** from treatment of [Au_2_(bis‐(2,6‐dimethylphenyl) formamidinate)_2_(NO_3_)_2_] **82** with KF.

In 2011, Reber and co‐workers prepared [Au_2_(bis(2,6‐dimethylphenyl)formamidinate)_2_(F)_2_], **83** (Figure 17).^[67]^ **83** was prepared by initially synthesising the dinuclear Au^(II)^‐nitrate precursor **82**, allowing the labile nitrate ligands to exchange with fluoride. Metathesis of **82** with KF led to the formation and crystallisation of **83**. The Au^(II)^‐F bond lengths (2.287 Å) in **83** are shorter than the heavier halides, such as Au^(II)^‐Cl bond (2.366 Å). **83** had significantly longer Au‐F bonds than other Au‐fluorides, such as [AuF_4_]^−^ (2.076–2.190 Å) and [(SIPr)AuF] (**3**) (2.028 Å),^[24]^ likely a result the strong *trans* influence in the Au^(II)^‐Au^(II)^ bond. The catalytic activity of **83** was not examined.

**Figure 18:**
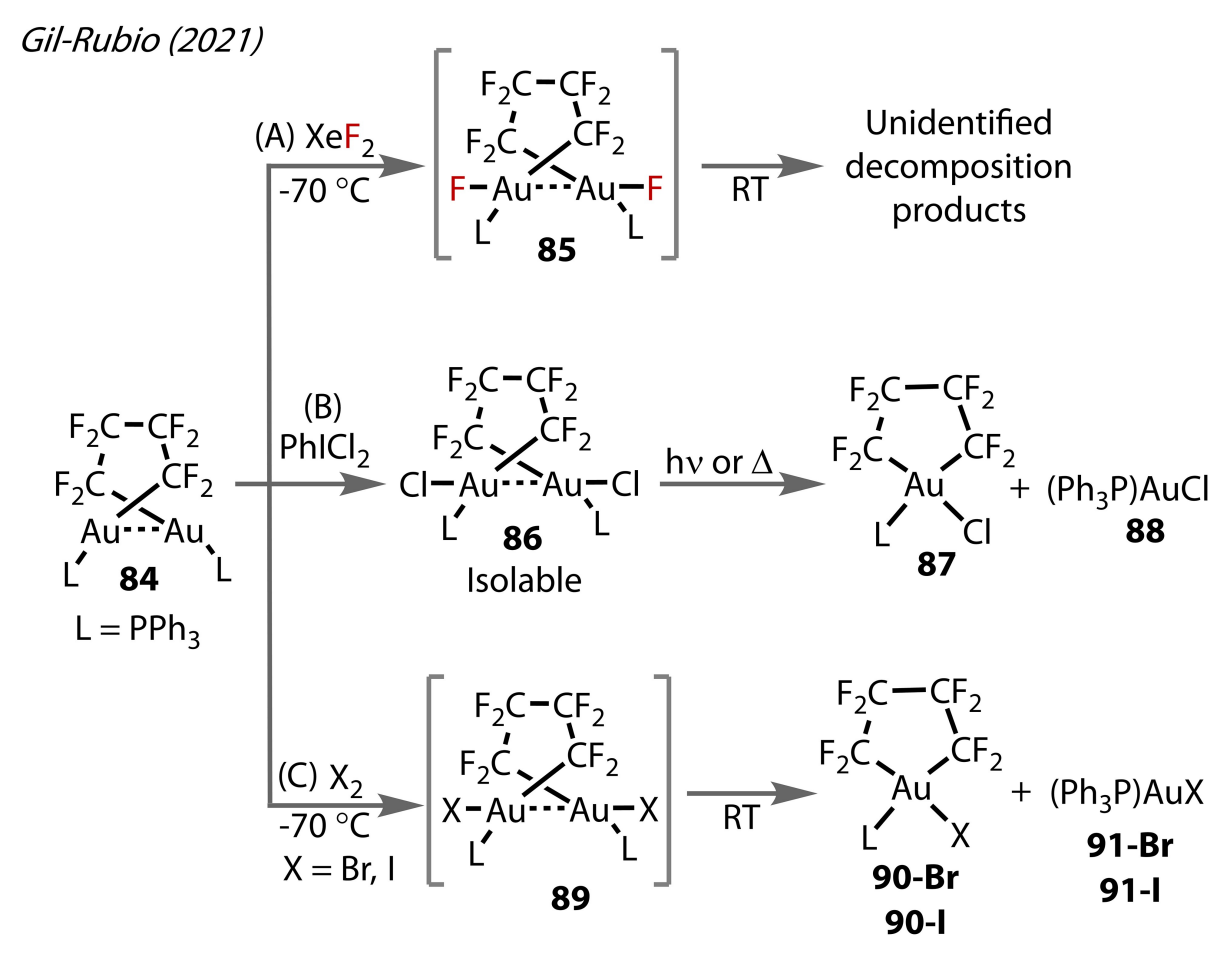
Gil‐Rubio‘s (2021) preparation of perfluoroalkyl‐tethered dinuclear Au^(II)^‐X halide complexes (X=F **85**, Cl **86**, Br **89‐Br**, I **89‐I**).

In a 2021 study examining the nature of aurophilic interactions using perfluoroalkyl‐tethered dinuclear gold complexes, Gil‐Rubio reacted a dinuclear Au^(I)^ complex **84** with XeF_2_ (Figure 18A),^[72]^ forming the Au^(I)^‐F complex **85**, indicated by a broad singlet in the ^19^F NMR at −186 ppm. This complex exhibited light and thermal sensitivity at room temperature. The mixture of decomposition products was not characterised. Attempts to access this species with other phosphine ligands were not detailed in this report. Contrastingly, PhICl_2_ oxidised **84** to the isolable chloride complex **86** (Figure 18B), converting to [Au{κ ^2^‐(CF_2_)_4_}Cl(PPh_3_)] **87** and Ph_3_PAuCl **88** when irradiated at 402 nm or heated to 80 °C for 1 hour. Similar instability to the fluoride complex was observed in the corresponding bromide and iodide complexes (Figure 18C), accessed by oxidation with Br_2_ or I_2_, and decomposing to [Au{κ ^2^‐(CF_2_)_4_}X(PPh_3_)] **90‐X** and Ph_3_PAuX **91‐X** (X=Br, and I respectively).

Organometallic Au^(II)^‐fluoride complexes are evidently scarce. Aside from the experimental examples already described, Toste (2011) has proposed a dinuclear Au^(II)^‐fluoride through DFT calculations as a catalytic intermediate in the oxidative intramolecular heteroarylation of alkenes.^[73]^

## 4 Au^(III)^‐Fluoride Complexes

Gold exhibits a relatively stable 3+ oxidation state, as opposed to copper and silver.^[74]^ Due to the increased hardness of Au^(III)^ relative to Au^(I)^, homoleptic AuF_3_ can be synthesised with greater ease than Au^(I)^‐F, and could be characterised in both gaseous and condensed phases. AuF_3_ was first accessed by Sharpe in 1949, who treated elemental Au with BrF_3_ and isolated solid AuF_3_ in a two‐step process.^[75]^ AuF_3_ is nevertheless very unstable, and subsequent reactions occur uncontrollably. Consequently, as seen for Au^(I)^‐fluorides, more stable organo Au^(III)^‐fluorides have been deployed, allowing for their reactivity to be extensively investigated.

### 4.1 NHC Ligands

**Figure 19:**
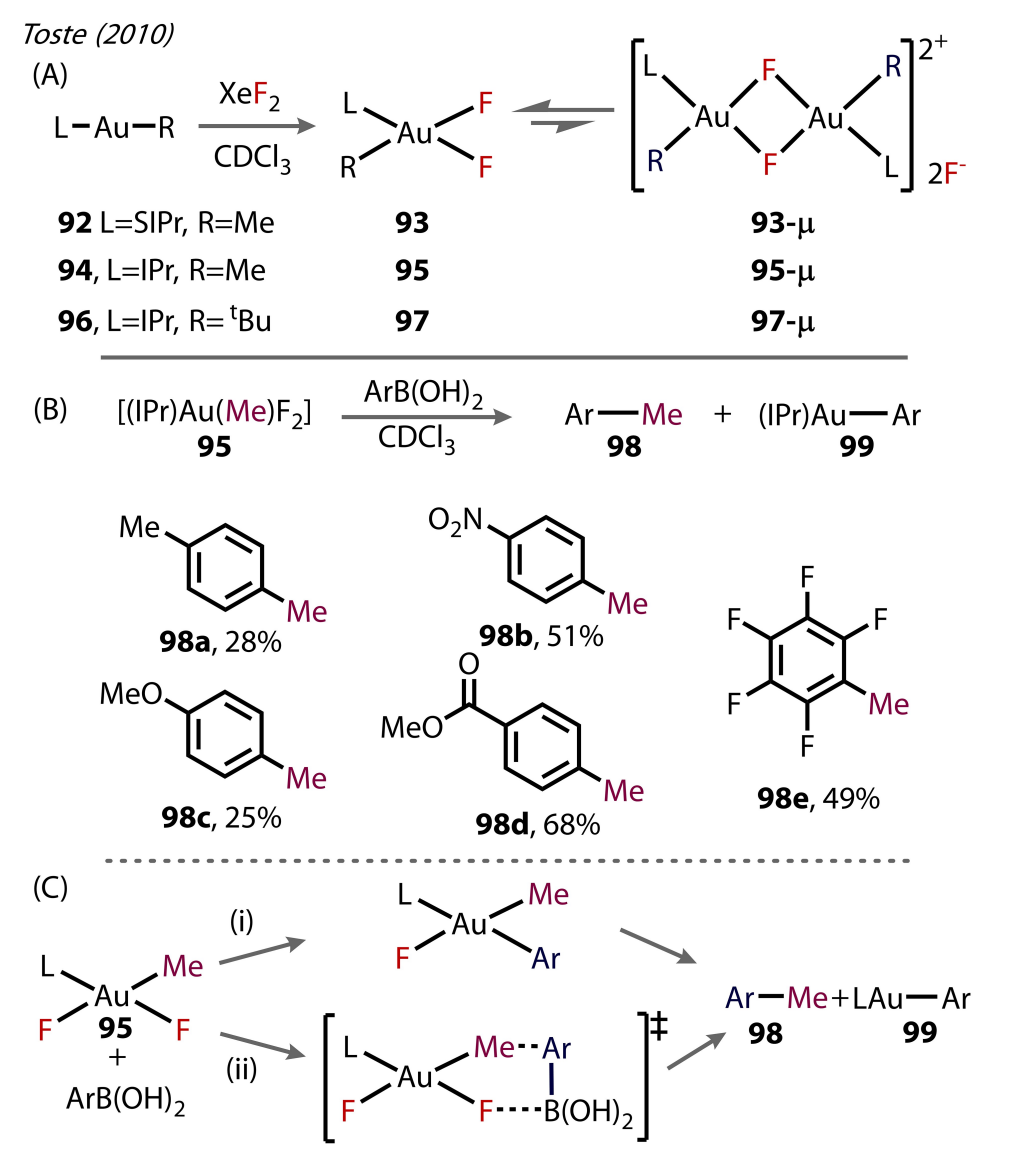
Toste (2010) study of NHC‐Au^(III)^‐F complexes. (A) Synthesis of cis‐Au^(III)^‐difluorides, with varying R‐groups. (B) The reaction of these complexes with boronic acids, yielding the C−C coupling products **98**. (C) possible pathways for C−C bond formation mediated by LAu(R)F_2_ species **95** R=Me, i) transmetalation followed by reductive elimination, or ii) bimolecular reductive elimination.

In 2010, Toste successfully utilized NHC ligands in a Au^(III)^‐fluoride complex (Figure 19).^[76]^ Initially, the authors attempted to fluorinate [(Ph_3_P)AuMe] with XeF_2_, but observed C_2_H_6_ and [(Ph_3_P)_2_Au]^+^. However, [(L)Au(R)], where either L=SIPr and R=Me (**92**), L=IPr and R=Me (**94**), or L=IPr and R=t‐Bu (**96**), all observed the cis‐Au(III)‐fluorides (Figure 19A) upon treatment with XeF_2_. The complexes formed were all characterised by NMR but only two dimers, **93‐μ** and **95‐μ**, were isolated in the solid state. Interestingly, **93** was exclusively favoured in solution, though it could only be crystallised as the dimer. For the *t*‐Bu derivative **96**, the bridged dimer **97‐μ** was observed exclusively, and was less stable than the less bulky dimeric complexes, decomposing after several hours in chloroform. The Au^(III)^‐F bond lengths of **93‐μ** were measured to be 2.034 Å for the bridged fluoride *trans* to SIPr, which was slightly elongated compared to [(SIPr)AuF], **3** (2.028 Å),^[24]^ likely a result of bridging. The fluoride *trans‐* to the Me, however, was much longer at 2.124 Å.

The structure of **95‐μ** is closely related to other proposed intermediates in C−C coupling reactions catalysed by Au^(I)^/Au^(III)^.[^77^, ^78^, ^79^, ^80^, ^81^, ^82^] The reactivity of the complexes towards various aryl‐boronic acids was subsequently explored (Figure 19B). The C_aryl_‐C_aryl_ coupling products were formed, with lower yields for more electron‐rich boronic acids attributed to oxidation of the aryl boronic acid. Notably, similar reactions with the analogous iodide complexes did not proceed, and instead rapidly decayed to [(IPr)AuI] and MeI by C−I reductive elimination. This comparison was key to highlighting the use of electrophilic fluorine oxidants in Au^(I)^/Au^(III)^ C−C coupling reactions, as the putative Au^(III)^‐F intermediates activate the boronic acid, in addition to being resistant to C−F elimination, contrary to the other halides. Further investigations of the mechanism considered two possible routes: (i) a two‐step transmetalation followed by reductive elimination, or, (ii) single‐step biomolecular reductive elimination (Figure 19C). A competition experiment was performed with a 1 : 1 mixture of an electron‐neutral and an electron‐deficient boronic acid with complex **95**, showing relatively little difference in reactivity, disfavouring pathway (i). Additionally, complex **97** was unreactive towards PhB(OH)_2_, where the opposite would be expected given the propensity of sterically encumbered ligands to undergo more facile reductive elimination. These observations suggest a bimolecular reductive elimination occurs in these complexes, as opposed to a direct transmetalation.

**Figure 20:**
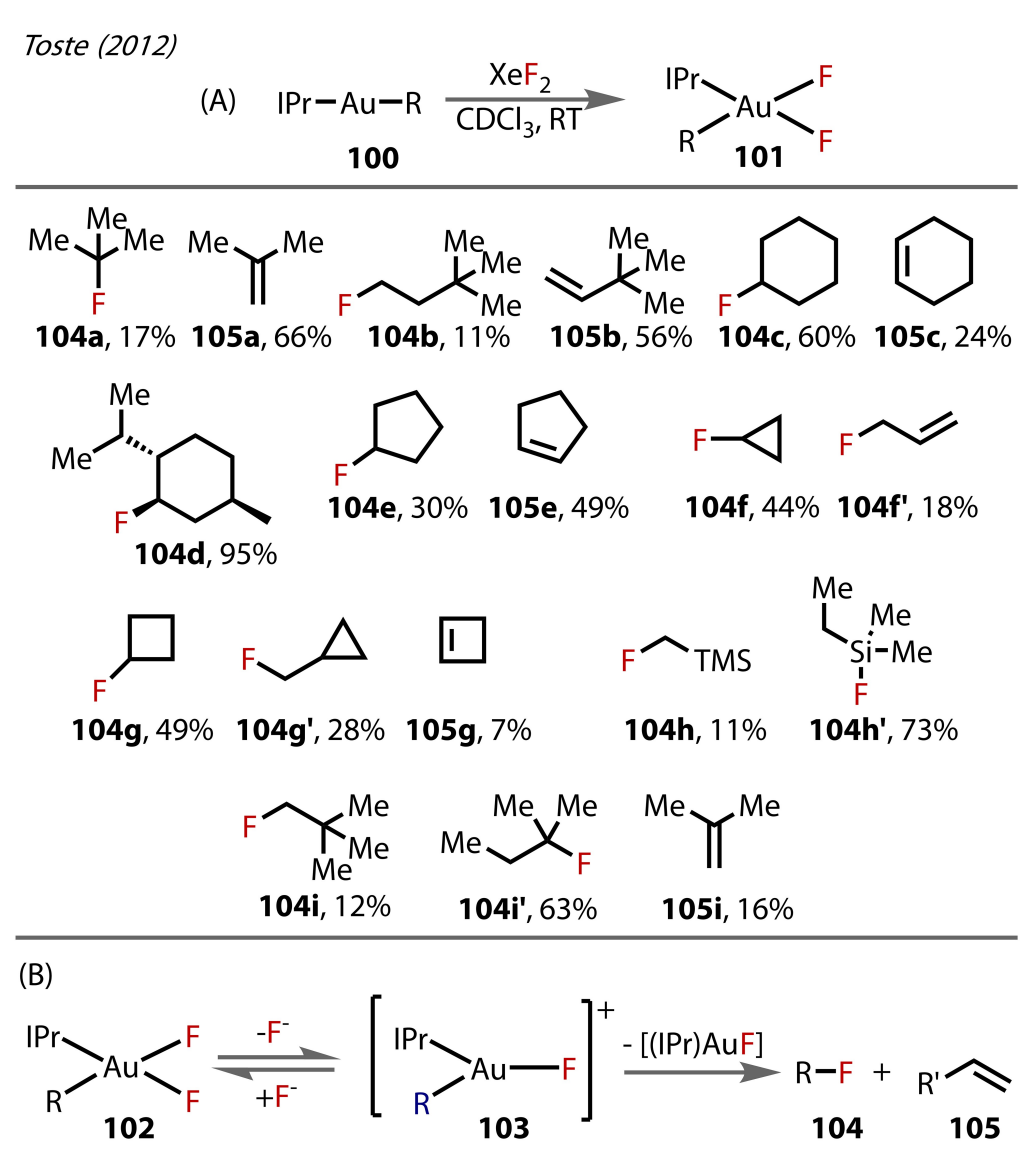
Toste's (2012) Au^(III)^ C(sp^3^)‐F reductive elimination study. (A) Substrate scope containing fluoroalkanes from C−F reductive elimination **104** and alkenes from β‐hydride elimination **105**. (B) Proposed mechanism of decomposition of Au^(III)^‐difluoride **102** via a 3‐coordinate intermediate.

In 2012, Toste and Mankad reported the preparation of a series of *cis*‐[F_2_Au(R)(IPr)] intermediates **101** (where R=alkyl) by oxidising the corresponding [(IPr)AuR] **100** with XeF_2_ (Figure 20A).^[83]^ Similar to the 2010 study,^[76]^ the complexes were observed by ^19^F‐ and ^1^H NMR, but not isolated in the solid‐state. The C(sp^3^)‐F reductive elimination pathway was investigated for **101**, forming [(IPr)AuF], as well as mixtures of alkenes from β‐H elimination and fluoroalkanes from C(sp^3^)‐F reductive elimination. Complexes possessing hindered R‐groups showed preference for β‐H elimination over C−F formation, with ratios 17 : 66 for **104** **a** : **105** **a** and 11 : 56 for **104** **b** : **105** **b**. This was likely the decomposition route for the unstable [(IPr)Au(^t^Bu)F_2_] in the previous 2010 study,^[76]^ which prevented its isolation.^[76]^ Cyclic alkyl groups were studied, with the prediction that β‐H elimination would be disfavoured, and C−F reductive elimination would be the major pathway. This was the case for **104** **c** : **105** **c**, which formed the fluoroalkane:alkene in a 60 : 24 ratio, and for **104** **d** where the fluoroalkane formed exclusively, in excellent yield. For the **104** **e** : **105** **e**, C−F reductive elimination was outcompeted by β‐H elimination (49 : 30). Strained 3‐membered and 4‐membered alkyl derivatives showed a preference for direct C−F reductives elimination, but minor pathways of carbocation‐rearrangements followed by C−F reductive elimination were also observed **104** **f/f**’ and **104** **g**/**104** **g’**/**105** **g**. Finally, groups lacking β‐hydrogens (**104** **h**/**h’** and **104** **i**/**i’**) were studied, showing a clear preference for C−F reductive elimination, with tertiary fluoroalkanes dominating over the expected primary isomers when methyl migration was possible.

Kinetic studies showed the decay of the Au^(III)^‐difluoride **102** to be inverse‐first order with respect to fluoride concentration. This observation, along with the isolation of the cationic dimeric complexes [(IPr)Au(F)(R)]_2_^+^ (**93‐μ**, **95‐μ**, **97‐μ**) from their 2010 study (Figure 19),^[76]^ rationalises an initial dissociation of the fluoride ligand to a 3‐coordinate intermediate **103** prior to reductive elimination (Figure 20B).^[76]^

As a comparison, the authors tested the reactivity of Au^(III)^‐iodides and found them to be much more reactive towards C−I reductive elimination. An unambiguous difference was in the methyl derivatives; the fluoride complex showed no C−F reductive elimination even at raised temperatures, unlike the Au^(III)^‐iodide, which exclusively form the iodoalkane in quantitative yields. Furthermore, rearrangements were also found to be limited compared to the corresponding Au^(III)^‐fluorides, suggesting the carbocationic character of alkyl‐Au^(III)^ complexes was heightened by fluoride ligands. This was also demonstrated with DFT calculations that showed the Au^(III)^‐F bonds to be ionic in nature, which in turn led to a build‐up of positive charge on the Au^(III)^ and polarising the Au‐alkyl bonds. The Au^(III)^‐iodide complex was never present in the NMR spectrum, unlike the Au^(III)^‐fluoride, which suggested the iodide analogues have very short life‐times, due to rapid C−I reductive elimination. The difference in the nature of the ionic Au^(III)^‐fluoride bonds compared to covalent Au^(III)^‐iodides gave rise to unique product distributions. Furthermore, unlike the Au^(III)^‐iodide, which underwent facile C−I reductive elimination, the fluoride analogues showed resistance towards this route.

**Figure 21:**
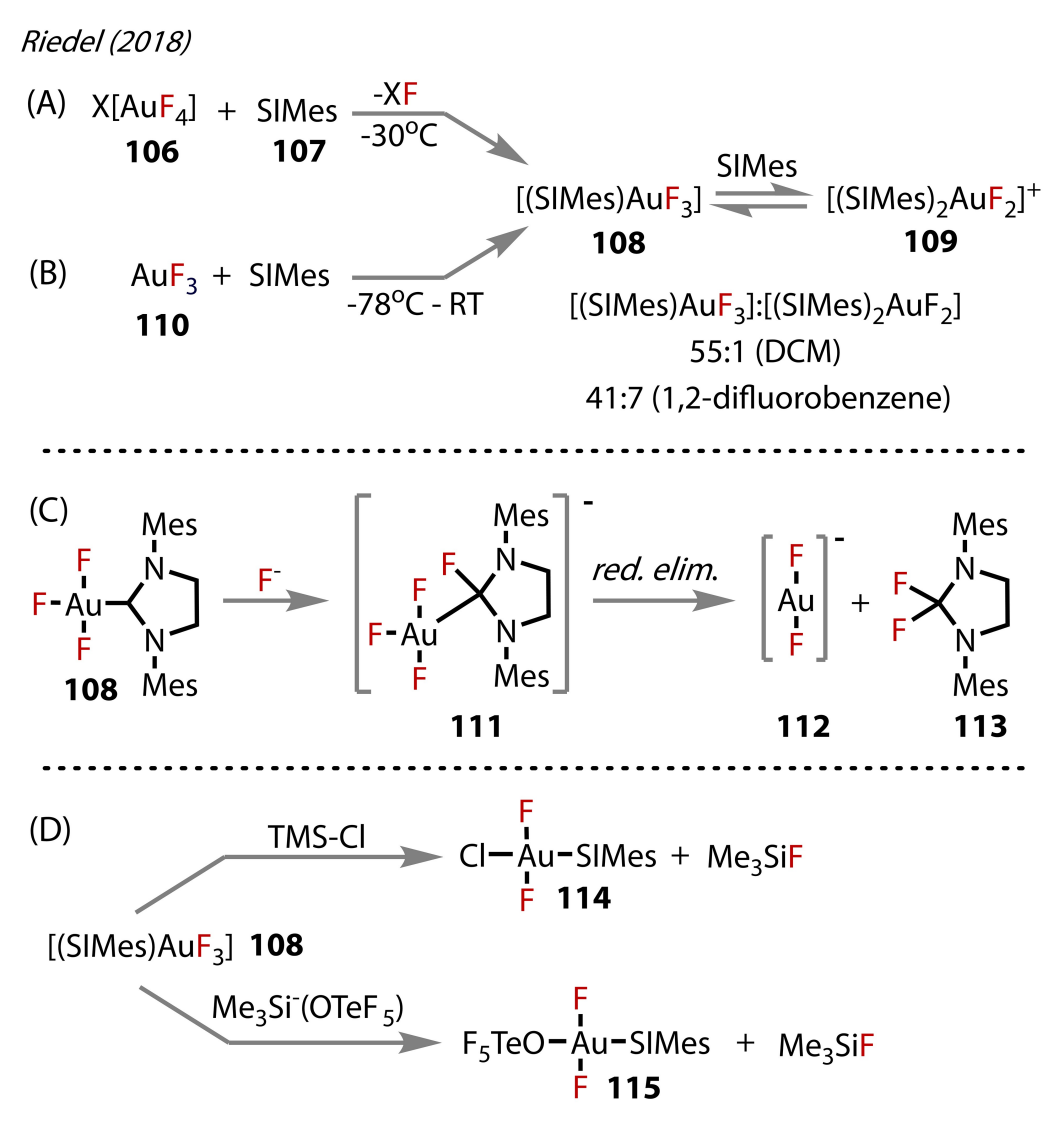
Synthesis of [AuF_3_(SIMes)] **108** by routes using: (A) X[AuF_4_] **106** where X=NMe_4_^+^ or Cs^+^. (B) AuF_3_ **110** in a either DCM or 1,2‐difluorobenzene. (C) Proposed mechanism for the fluorination of SIMes. (D) Metathesis of **108** with TMSCl and TMS‐teflate.

The other NHC ligand known to stabilise Au^(III)^‐fluorides is SIMes=1,3‐bis(2,4,6‐trimethylphenyl)‐4,5‐dihydro‐imidazol‐2‐ylidene, which was reported in 2018, by Riedel, who developed two routes to [AuF_3_(SIMes)] (**108**).^[84]^ Both routes resulted in products that were isolated; this stability was exceptional, as it was the first Au trifluoride complex to be characterised in the solid‐state. The first method followed treatment of [X][AuF_4_] (X=NMe_4_^+^ or Cs^+^) **106** with SIMes **107** in DCM (−30 °C), yielding both the neutral **108** and, simultaneously, [(SIMes)_2_AuF_2_]^+^ (**109**) (Figure 21A). The [AuF_2_]^−^ by‐product **112** is assumed to form, but was never characterised, presumably due to its high instability in condensed‐phase. Alternatively, **108** was synthesised from treatment of AuF_3_ **110** with SIMes in a suitable solvent. The solvent choice was significant, and, after screening, DCM and *ortho*‐difluorobenzene (−78 °C) were found to be most selective towards **108** (55 : 1 and 41 : 7 respectively for species **108** : **109**) (Figure 21B). SIMesF_2_ was also present in the reaction solution, presumably from fluoride attacking the SIMes ligand of **108** and reductive elimination (Figure 21C).

The X‐ray crystal structure of **108** showed the Au^(III)^‐F bond *trans* to SIMes (1.972 Å) was elongated compared to those *cis* to SIMes (1.916 and 1.921 Å), due to SIMes’ *trans* influence. The authors noted the *cis* positioned Au^(III)^‐F bonds were close to those in the [AuF_4_]^−^ complex (1.908 Å). Whereas *trans* fluorides were still shorter than the Au^(I)^‐F bonds *trans* to NHC ligands, reflecting the increased stability of Au^(III)^‐fluorides relative to Au^(I)^‐fluorides. The high Lewis acidity of **108** was reflected in the shorter Au^(III)^‐C bonds, and correspondingly elongated *trans‐*Au‐F bond.

The increased Lewis acidity of the fluoride complexes compared to chloride, coupled with the more labile *trans*‐Au^(III)^‐F, relative to *cis*‐positioned fluorides, opened up possibilities of reactions with these complexes (Figure 21D).^[85]^ From **108**, two more novel Au^(III)^‐fluoride complexes were isolated and characterised by X‐ray crystallography. The first complex, [AuClF_2_(SIMes)] **114** was accessed from **108** with Me_3_SiCl in DCM (Figure 21D), resulting in chloride exchange with the fluoride *trans* to the SIMes ligand. Elongation of the Au‐F bond resulted in increased reactivity, as illustrated by only the *trans*‐positioned fluoride reacting, even in excess Me_3_SiCl. [AuF_2_(OTeF_5_)(SIMes)] **115** was synthesised using excess Me_3_Si(OTeF_5_) with **108**. The choice to add teflate to this type of complex was due its efficient leaving group ability, thereby presenting as a promising candidate for precursors to other Au‐fluoride complexes. **114** and **115** have Au^(III)^‐F bond lengths of 1.920–1.931 and 1.891–1.944 Å, respectively, comparable to the Au^(III)^‐F of **100** (1.916 and 1.921 Å). The authors calculated SIMes affinities (i.e. the ΔG for carbene dissociation) for the [AuF_2_X] species, which correlated with the carbene ^13^C NMR chemical shift: a more up‐field signal correlated with a higher SIMes affinity, and a higher Lewis acidity. The ^13^C NMR peaks for the carbene carbon of the Au^(III)^‐F complexes showed a down‐field shift for **114** compared to **108** (166.0 vs 152.4 ppm), suggesting **108** had a more Lewis acidic Au^(III)^‐centre. In contrast, **115** was up‐field shifted (149.4 ppm), correlating with higher Lewis acidity than **108** and **114**. This trend aligns with the differing *trans*‐influence in the order of Cl>F>OTeF_5_, with a stronger *trans*‐influence weakening the Au‐carbene bond, resulting in a more deshielded carbene environment in the ^13^C NMR.

**Figure 22:**
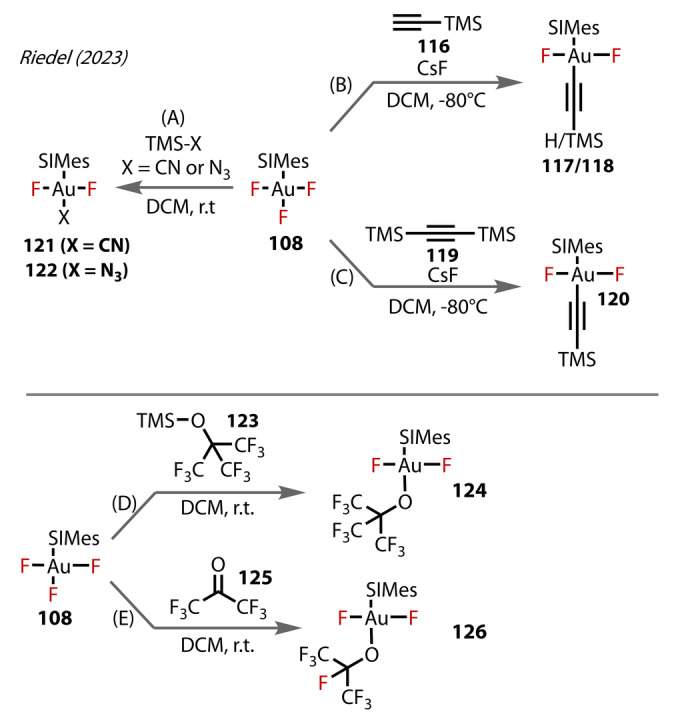
Riedel's (2023) reactivity study on [(SIMes)AuF_3_] **108**. (A–C) Transmetalation with organosilicon coupling partners. (D, E) Formation of alkoxide complexes **124** and **126**.

The significance of Riedel's study arises from the ability of SIMes ligands to stabilise very Lewis acidic Au^(III)^‐fluorides, which have potential applications in fluorination reactions, as demonstrated in their subsequent 2023 study. [AuF_3_(SIMes)] **108** was reacted with TMS‐X (X=CN, N_3_) Figure 22A), affording the monosubstituted products, *trans*‐[Au(CN)F_2_(SIMes)] **121** and *trans‐*[AuF_2_(N_3_)(SIMes)] **122**. Triple substitution occurred with an excess of the TMS reagent, with the double substitution product not observed. At −80 °C, the Au‐alkynyl species **117** and **118** Figure 22B and C), was formed by reaction with TMS‐acetylene (**116**), giving rise to a mixture of the C‐[Si] activation products **117** and C−H **118**, with only single substitution observed. The presence of CsF favours the formation of the C‐[Si] activation products. **120** was also accessed through reaction with the bis‐TMS alkyne **119**, retaining the TMS group. The authors synthesised O‐ligated complexes; room‐temperature reaction with TMS‐OC(CF_3_)_3_ **123** yielded *trans*‐[AuF_2_(OC(CF_3_)_3_)(SIMes)] **124**,^[86]^ Figure 22D/E). The [AuF_3_(SIMes)] could insert electron‐deficient carbonyl **125** to form the corresponding perfluoroalkoxido‐Au^(III)^ complex **126**. The authors correlated the δ(^13^C_carbene_) with the SIMes affinity and the Au‐C_carbene_ bond lengths. The various ligands in the series could then be classified according to their *trans‐*influence OTeF_5_<F<OCF_3_<OC_2_F_5_<OC_3_F_7_<OC_4_F_9_Cl< CN≈N_3_<CCH≈CCSiMe_3_<CF_3_. The higher the *trans*‐influence in this series corresponding to more electron‐withdrawing ligands, the shorter the Au‐carbene bond. This trend applies to both the solid‐state and in solution.

### 4.2 Phosphine Ligands

**Figure 23:**
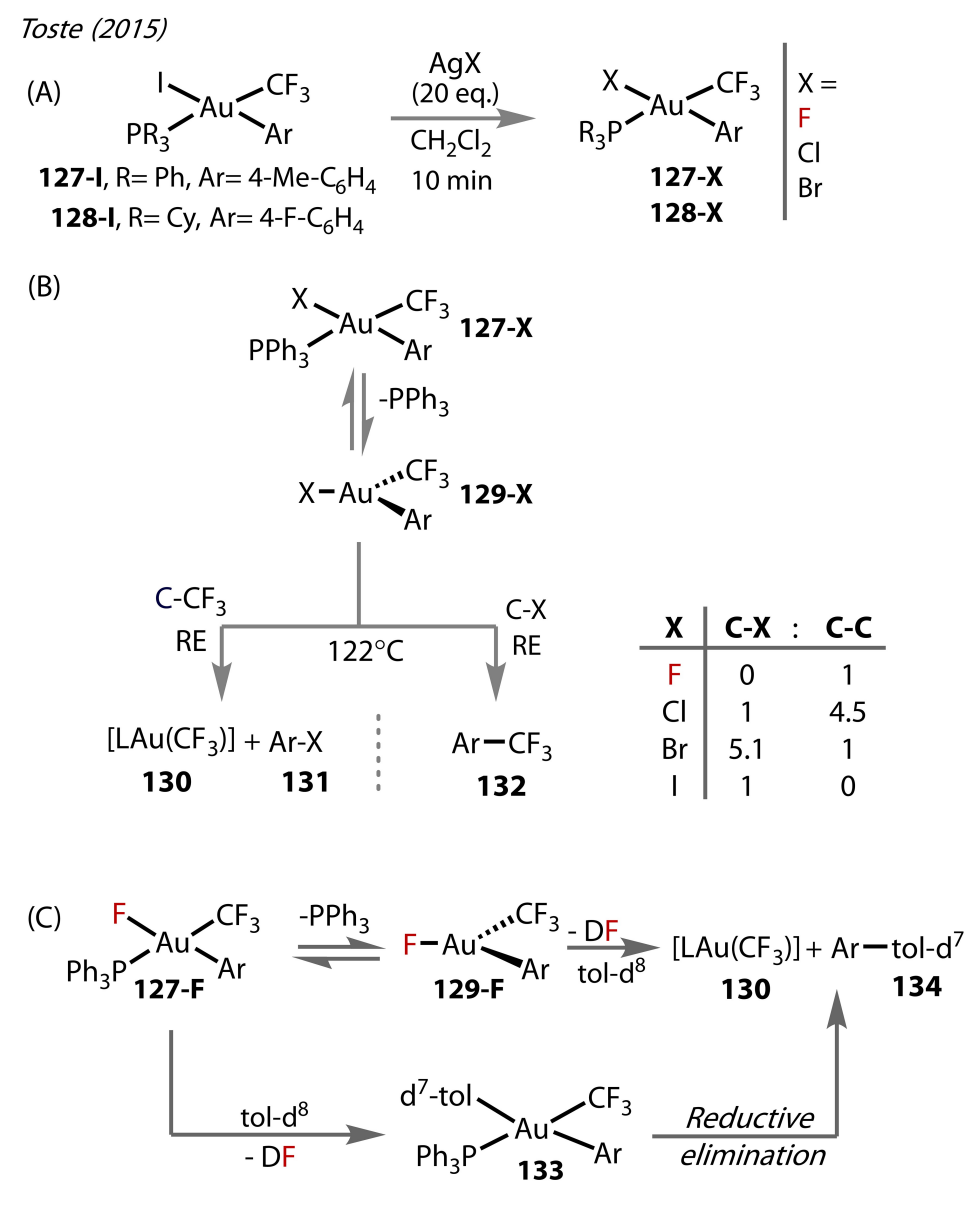
Toste (2015). (A) Synthesis of Au^(III)^‐halides, **127/128‐X** supported by phosphine ligands from I/X exchange with AgX X=F, Cl, Br. (B) Proposed mechanism of C_aryl_‐X and C_aryl_‐CF_3_ reductive elimination of **127‐X**, where X=I, Br, Cl, F and product ratios. (C) Solvent activation of [(PPh_3_)Au(Ar)F(CF_3_)] with toluene, followed by reductive elimination of the activated species to give C_aryl_‐C_aryl_ **134** via a 3‐coordinate intermediate **129‐F**, or by Au‐Ar complex **133**.

In 2015, Toste and co‐workers, reported the synthesis of a series of halide complexes of the type [(R_3_P)Au(aryl)(CF_3_)(X)] (X=Br, Cl, F) (**127‐X** and **128‐X**) through halide‐metathesis of [(R_3_P)Au(aryl)(CF_3_)(I)] (**127‐I** and **128‐I**) with AgX (X=F, Cl, Br) (Figure 23A).^[87]^ The series included one of the first examples of a complex with terminal Au^(III)^‐F that were isolable in solid‐state.

Thermolysis of **127‐X** yielded products from both C_aryl_‐X (X=I, Br, Cl, F) **131** reductive elimination and C_aryl_‐CF_3_ **132** reductive elimination with varying selectivity depending on the halide (^19^F NMR) (Figure 23B). The authors reported differences in the thermolysis rate for the halides, with **127‐F** being slower than **127‐I** (t_1/2_=33 vs 2.5 min) but faster than both **127‐Cl** and **127‐Br** (t_1/2_=75 and 400 min, respectively). Similarities between **127‐F** and **127‐I** were also observed, both showing zero‐order kinetics. The rates then became inverse‐first order in relation to phosphine concentration, when phosphine was added, which suggested the reaction proceeded via phosphine dissociation to a 3‐coordinate intermediate, **129‐X**.

There was an overall trend in the preference for each reductive elimination route for the halides. **127‐F** showed exclusive selectivity for C_aryl_‐CF_3_ reductive elimination, with decreasing selectivity towards C‐CF_3_ and increasingly towards C−X reductive elimination down the group, with **127‐I** exclusively forming the C−I reductive elimination product. The study highlighted the stark difference in the preference for coupling between Au^(III)^‐fluoride and Au^(III)^‐iodide. This trend aligns with DFT studies from Datta (2017) who calculated the relative reductive elimination pathways of these gold halide complexes,^[88]^ and provides support for the use of Au^(III)^‐fluorides in oxidative coupling reactions for C−C instead of C−F bond formation.

When studying the thermolysis of **127‐F**, the authors discovered that the C‐CF_3_ reductive elimination also competed with activation of the toluene‐*d* ^*7*^ solvent (Figure 23C), based on the generation of biaryl‐*d* ^*7*^ **134**. Further studies showed solvent activation was independent of phosphine; the authors reasoned that this was plausible because **127‐F** was sufficiently Lewis acidic to activate the C−D bonds.

**Figure 24:**
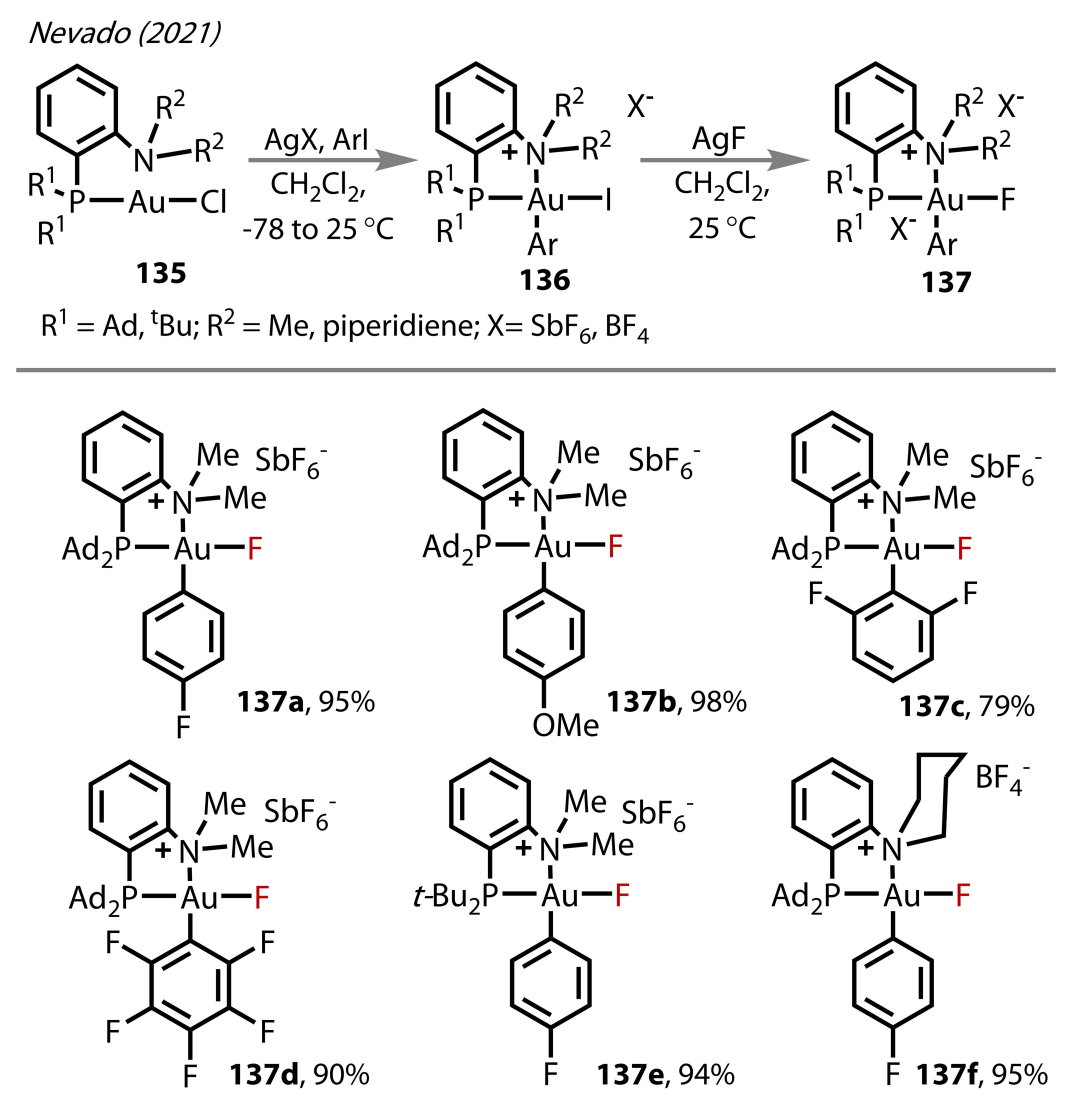
Nevado‘s (2021) synthetic route to (P N)Au‐F complexes **137**, and substrate scope.

A family of P N stabilised Au^(III)^‐fluorides employing the DalPhos ligand family was reported by Nevado and co‐workers in 2021, bearing the previously unreported *trans*‐P‐Au‐F (Figure 24).^[89]^ Six complexes were prepared from the Au^(I)^‐Cl complex **135** via initial oxidative addition of Ar‐I to (P N)Au^(III)^‐iodide **136**, followed by F/I exchange with AgF to yield **137**. The complexes were stable in solution for two days and most (**137** **a**, **b**, **d**, **e**) were isolable as solids. X‐ray crystallography analysis observed all isolated complexes in monomeric form exhibiting a distorted square planar geometry. The large P−F coupling constant (^2^ *J*_P,F_=100.5 Hz) aligns with this *trans* P−Au‐F arrangement. The Au^(III)^‐F bond lengths were in the range 2.006–2.133 Å. Interestingly, the bond lengths increased with increasingly electron‐poor aryl substituents: (*p*‐F): 2.023 Å, (*p*‐OMe): 2.006 Å; (pentafluoro‐): 2.133 Å). Due to difficulty in obtaining crystals of **137** **f**, the structure was examined through DFT calculations (B3LYP/6‐31G(d,p), SDDAll(Au), SMD=dichloromethane). The calculated bond length was comparable to **137** **a** and **137** **e**, implying the aryl ligands had a larger influence on the Au^(III)^‐F bond length compared to P‐ and N‐ counterparts, but that the stability of the complex was influenced by N‐substituents. In the case of **137** **f**, the increased steric demand of the N‐ligand resulted in a more ‘disrupted’ square planar geometry (a smaller C−Au‐F angle of 80.1° versus 84.3° in its corresponding NMe_2_ complex), an increased electropositive Au^(III)^‐centre (δAu=+0.75 versus 0.724) and in turn, a more polarised Au^(III)^‐F bond.

**Figure 25:**
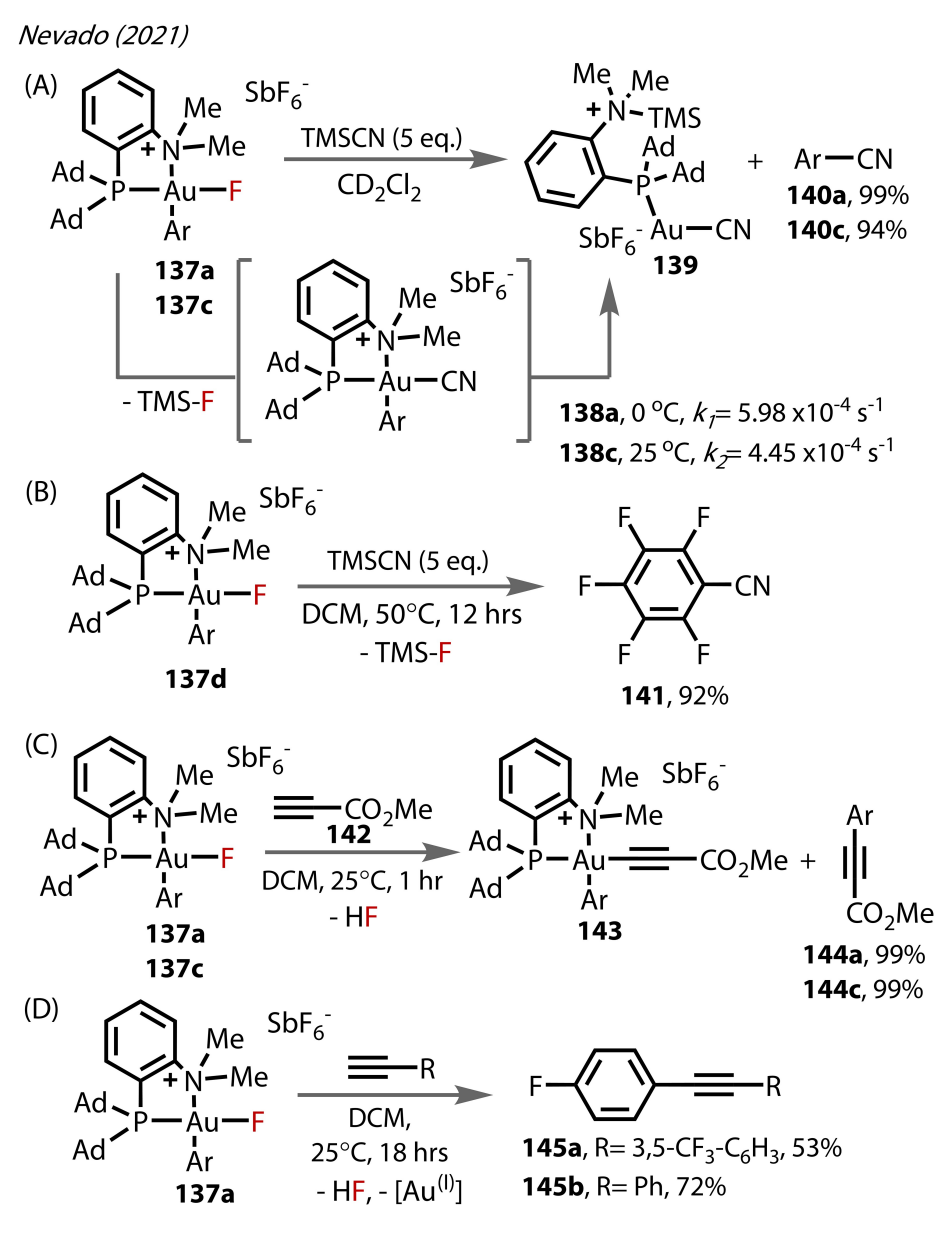
Nevado's (2021) study on stoichiometric P N‐Au^(III)^‐F **137** reactivity. (A) Ar‐CN coupling to form **140**, (B) C−N coupling with an electron‐deprived aryl group forming **141**. (C) C−C coupling with a propionic ester **142** to form internal alkynes **144**. (D) C−C coupling with aryl acetylenes to form internal alkynes **145**.

Whilst Au^(III)^‐fluoride complexes had facilitated sp^3^ and sp^2^ hybridised C−C reductive eliminations,[^83^, ^90^] C(sp^2^)−C(sp) reductive elimination from these complexes had not been previously reported. Reactions of **137** **a** and **137** **c** with TMSCN were conducted, undergoing transmetalation to form **138** **a** and **138** **c**, eventually yielding **140** **a** and **140** **c** quantitively by reductive elimination alongside **139** (Figure 25A); whereas the very electron‐poor **141** (Figure 25B) could only form at elevated temperatures from **137** **d**. This could be considered unusual given the increased bond length of the more electron‐deprived Au^(III)^‐fluorides, but shows the significant impact of sterics on nitrogen. Similar reactivity was also observed for alkynes, producing C−C coupling products from **137** **a** and **137** **c** (forming **144** **a** and **144** **c**) using propionic ester **142** (Figure 25C). Arylacetylene substrates were slightly lower yielding (**145** **a** and **145** **b**) (Figure 25D). The rates of reductive elimination exceeded those of other Au^(III)^‐phosphines.^[91]^

**Figure 26:**
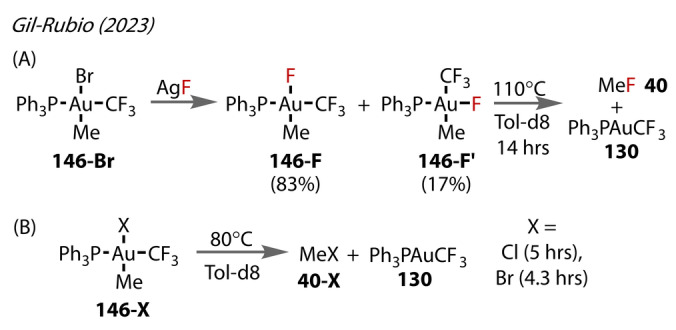
Gil‐Rubio's (2023) C−X reductive elimination from Au^(III)^ phosphine complexes **146‐X**. (A) C−F reductive elimination to form MeF from **146‐F** requiring a longer reaction time and higher temperature to reach full conversion than the corresponding chloride and bromide**146‐Cl/Br** complexes in (B).

Gil‐Rubio (2023) prepared methyl Au(III)‐F complex **146‐F** bearing a triphenyl phosphine ligand through metathesis of the bromide complex with AgF (Figure 26),^[92]^ as described in Toste's 2015 study.^[87]^ The thermal reductive elimination pathways for this complex were studied, along with the other halides. Interconversion between the *cis‐* and *trans*‐ isomers **146‐F** and **146‐F’** occurred throughout the process. C−F reductive elimination from these complexes to form MeF **40** was observed, requiring heating to 110 °C for 14 hours. Conversely, the bromide and chloride complexes **146‐Cl** and **146‐Br** required much milder conditions to undergo C−Cl and C−Br reductive elimination, achieving full conversion after 4–5 hours at 80 °C. The relative reluctance towards C−F reductive elimination compared to the other halides is in alignment with Toste's work. CF_3_‐X reductive elimination products were not observed in any case.

**Figure 27:**
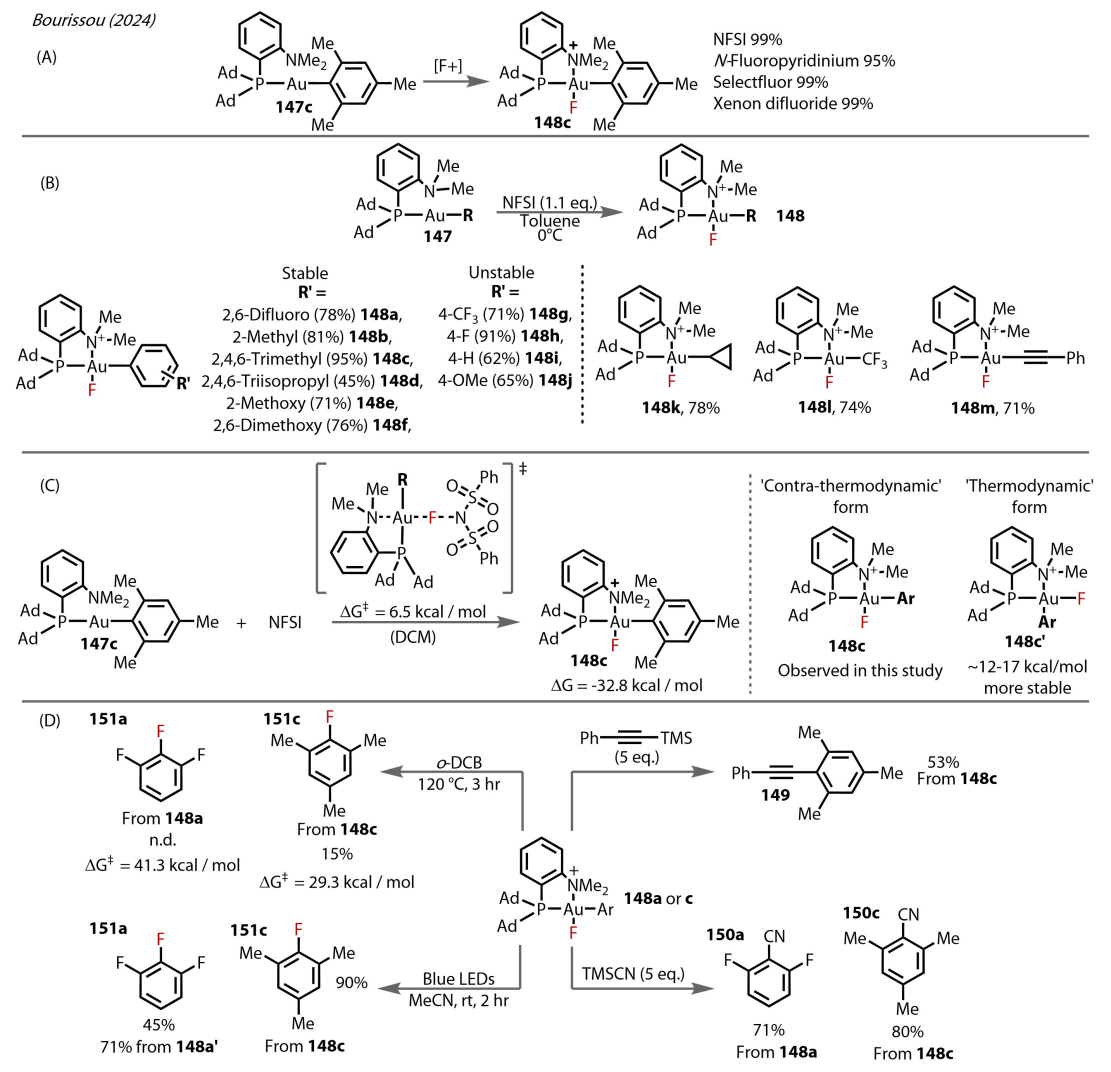
Bourissou (2024). (A) P N‐Au^(III)^‐F complex **148** formation using electrophilic fluorine‐based oxidants on **147**. (B) Scope of the R group *cis* to the fluoride ligand. (C) Computational analysis on the fluorination process and the differences between the *cis‐* **148** **c** and *trans‐* **148** **c’** isomers. (D) Bottom, clockwise: C−C **149**, C−N **150** and C−F coupling **151**. C−F coupling is enabled by irradiation with blue LEDs, whereas thermal C−F reductive elimination is low yielding.

In 2024, Bourissou reported an extension of their studies on P,N‐bidentate Au^(III)^‐F complexes by employing electrophilic fluorine‐based oxidants with the corresponding alkyl‐, aryl‐, and alkynyl‐ substituted MeDalphos‐Au‐R complex (Figure 27).^[93]^ The authors examined various oxidants, which each enabled rapid conversion of Au(I) phosphine **147** **c** to the Au^(III)^‐F complex **148** (Figure 27A). Regarding the scope of this protocol (Figure 27B), *ortho*‐substitution at the aryl ligand is necessary to afford stability to these Au^(III)^‐fluoride complexes (**148** **a**–**f** stable compared to **148** **g**–**j**), whereas varying the electronics of this aryl group had no effect. Alkyl‐ (**148** **k** and **l**) and alkynyl‐ (**148** **m**) substituted complexes are also stable, despite lacking steric bulk. Regarding the NFSI oxidation of **148** **c**, a low activation barrier was calculated at ΔG^≠^=6.5 kcal/mol (Figure 27C). Compared to similar complexes, with the fluoride ligand trans to the phosphine, the Au‐F bond length is 0.1–0.2 Å shorter (1.903 to 1.941 Å). This difference is reflected in the smaller trans‐influence of N than P. Accordingly, the experimentally observed contra‐thermodynamic form **148** **c** was less stable according to their DFT calculations. The energy gap between these two forms ranged from 12.2 to 16.9 kcal/mol, and was virtually unchanged with respect to sterics and electronics.

Reaction of **148** **c** with 1‐phenyl‐2‐trimethylsilylacetylene at 80 °C yielded the internal alkyne **149** (Figure 27D). Cyanation with TMS‐CN proceeded at room temperature in high yields from the mesityl and 2,6‐difluorophenyl complexes **150** **a** and **150** **c**. C−F reductive elimination proved difficult, as the complexes **148** **a** and **148** **c** decomposed after 3 hours, only resulting in a low yield of the aryl‐fluorides **151** **a** and **151** **c** after heating, in alignment with their calculated activation energies of 29.3 and 41.9 kcal/mol respectively for their thermal reductive eliminations. The authors considered irradiation of the Au^(III)^‐fluoride complex to induce reductive elimination, considering the success of this strategy in various cross‐coupling reactions.[^94^, ^95^, ^96^, ^97^] Complex **148** **c** exhibits a broad absorption peak at λ_max_ ∼327 nm which, based on DFT calculations, corresponds to the HOMO–LUMO excitation. The HOMO mainly consisting of the mesityl π‐system, and the LUMO comprising the Au‐N and Au‐F σ* anti‐bonding orbitals. Accordingly, irradiation of this complex with blue LEDs resulted in C−F reductive elimination and a very high yield of the mesityl fluoride **151** **c** at room temperature. 1,2,3‐trifluorobenzene **151** **a** was also able to form from **148** **a** in 45 % yield (and in 71 % yield from the “thermodynamic” isomer of the Au^(III)^‐F complex **148** **a’**).

It is evident that Au‐F transmetalation provides many avenues for future exploration in method development using a Au(I)/Au(III) redox catalysis approach. Bourissou's work, though a stoichiometric proof‐of‐concept, undoubtedly sets the stage for such reactivity by achieving difficult C−F reductive elimination under mild conditions—both in the ability to employ relatively mild electrophilic fluorine oxidants (NFSI, *N‐*fluoropyridinium) due to the electron‐rich bidentate ligand structure, and in the rapid room temperature reductive elimination step under blue LED irradiation.

### 4.3 Trifluoromethyl Ligands

**Figure 28:**
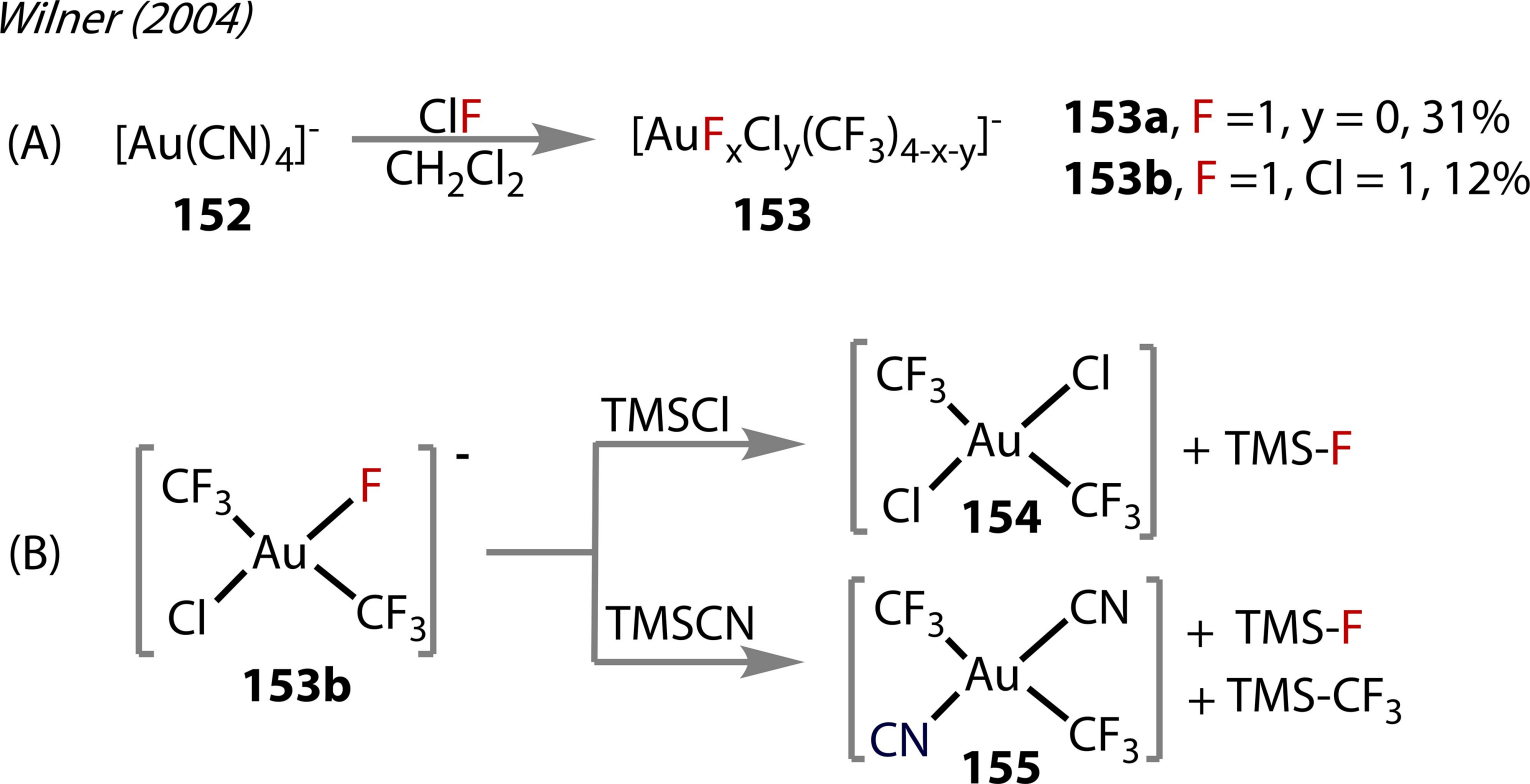
Wilner's (2004). (A) synthesis of Au^(III)^‐fluorides **153** from addition of ClF . (B) Exchange pathways of *trans*‐[F‐AuCl(CF_3_)_2_]^−^ **153** **b** with TMS‐Cl and TMS‐CN.

The first Au^(III)^‐fluorides possessing trifluoromethyl ligands were synthesised by Willner in 2004 (Figure 28),^[98]^ through a multistep process, which began with initial formation of [Au(CN)_4_]^−^, **152**. Fluorination with ClF, yielded a mixture of complexes of the form [AuF_x_Cl_y_(CF_3_)_(4‐x‐y)_]^−^ (*x*=0–4, y=0–2), **153** **a** and **153** **b** (Figure 28A). **153** **a** was found to be the most abundant Au^(III)^‐fluoride complex (31 %) yield, followed by **153** **b** (12 %). The complexes were identified in solution by NMR but were not isolated in their solid‐state.

The authors reported difficulties when attempting to characterise the Au^(III)^‐fluoride complexes by ^19^F NMR, with them exhibiting hydrolytic instability. The hydrolysis reaction reportedly released chlorides from chloride products in the mixture, which subsequently underwent F/Cl exchange with the Au^(III)^‐F bonds.. Furthermore, in DCM‐*d* ^*2*^, slow Cl/F exchange occurred, retaining *trans‐*stereoselectivity. More exchange reactions were tested, demonstrating the lability of the Au^(III)^‐F bond. **153** **b** was treated with (Me_3_)_3_SiCl and underwent Cl/F exchange with retention of *trans‐* stereochemistry (Figure 28B). Similar results were obtained with (Me_3_)_3_SiCN, which showed the reactivity of Au‐fluoride was governed by the higher affinity of Au for cyanide and chloride than for fluoride.

**Figure 29:**
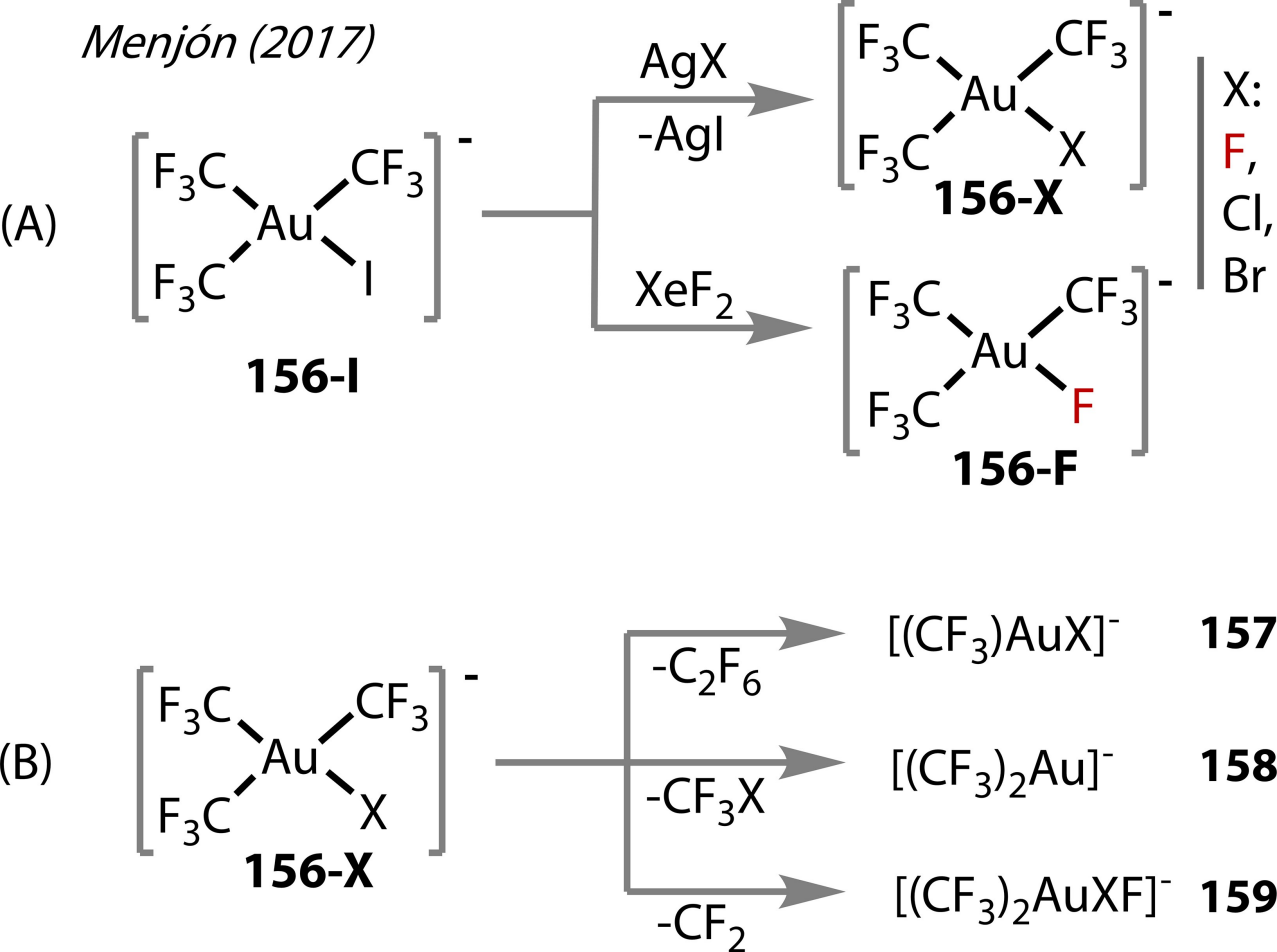
Menjón (2017). (A) Routes to [(CF_3_)_3_AuX] **156‐X** with AgX, X=F, Cl, Br and [(CF_3_)_3_AuF] **156‐F** using XeF_2_, from [(CF_3_)_3_AuI] **156‐I**. (B) Intramolecular decomposition paths of [(CF_3_)_3_AuX] **156‐X** X=F, Cl, Br, I.

In 2017, Menjón reported two routes to [(CF_3_)_3_AuF]^−^ **156‐F** (Figure 29),^[56]^ which was isolated in the solid state. The first method involved treatment of [(CF_3_)_3_AuI]^−^, **156‐I**, with AgF, where the stable AgI by‐product drives Au^(III)^‐F bond formation (Figure 29A). An alternative route with XeF_2_ was detailed, undergoing similar I/F ligand exchange. In solution, the anionic complex **156‐F** was extremely reactive towards trace water, but contrastingly was very stable in solid‐state, reportedly decomposing at 267 °C, which is higher than iodide‐analogues at 250 °C.

Unimolecular decomposition of [(CF_3_)_3_AuX]^−^, **156‐X** X=F, Cl, Br, I in gas‐phase was studied (Figure 29B). The fluoride‐complex showed clear differences to the heavier halides, mostly undergoing CF_2_ extrusion to [(CF_3_)_2_AuF_2_]^−^ **159**. Observed for all halides was the formation of [(CF_3_)AuX]^−^ **157** by C_2_F_6_ elimination, followed by a second decomposition (extrusion of CF_2_) to Au^(I)^‐fluorides [FAuX]^−^ X=F, Cl, Br, I.

**Figure 30:**
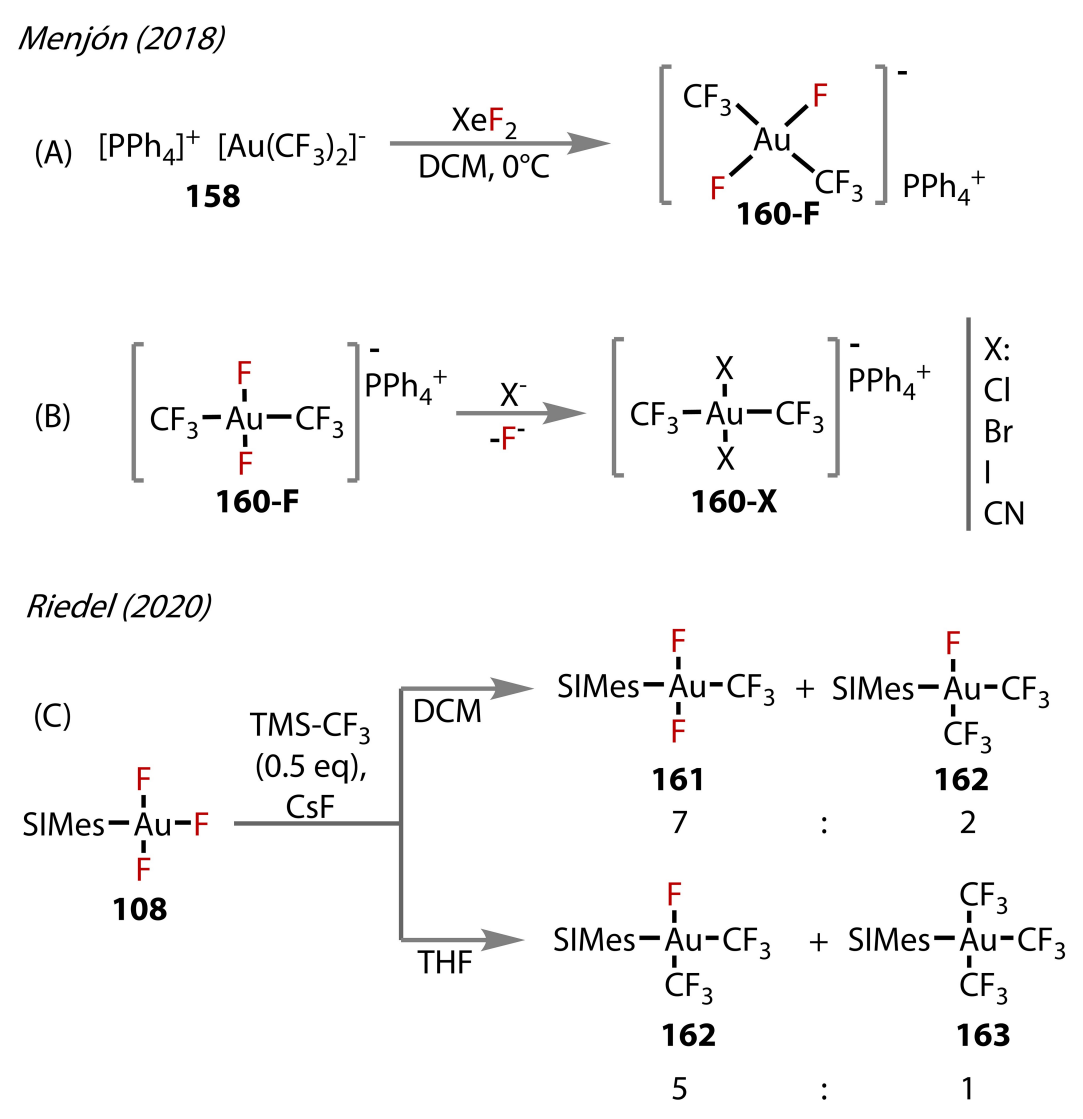
(A) Synthetic route to the *trans‐*[(CF_3_)_2_AuF_2_]^−^ anionic complex **160‐F**. (B) Synthesis of [(CF_3_)AuX_2_]^−^, X=Cl, Br, I, CN (**160‐X**) from ligand exchange of the fluoride from complex **160‐F**. (C) Preparation of Au^(III)^‐CF_3_ complexes (**161**–**163**) from SIMes‐Au^(III)^‐F_3_ **108**.

In 2018, a route to thermally stable and reactive [*trans*‐(CF_3_)_2_AuF_2_][PPh_4_] salts **160‐F** was developed by the same authors (Figure 30).^[99]^ Its synthesis employed again XeF_2_, however in this case undergoing oxidative addition to [(CF_3_)_2_Au][PPh_4_], to produce **160‐F** both quantitively and stereoselectively. The complex was of significance, being the first Au^(III)^‐difluoride complex characterised with fluorides in a *trans* arrangement, along with Dutton's N‐coordinated *trans‐*difluoro complexes reported in the same year,^[100]^ *vide infra*. The Au^(III)^‐F bond lengths were comparable to other Au^(III)^‐fluorides at 1.99 Å.[^90^, ^101^]

The reactivity of **160‐F** was investigated with heavier halides, X^−^ (X=Cl, Br, I) and cyanide, showing rapid exchange with fluoride and retention of *trans‐*stereochemistry for all ligands (**160‐X**), in good yields (Figure 30B).

Trifluoromethyl Au^(III)^‐fluorides up to this point had all been generated from trifluoromethyl ligated Au‐complexes, but in 2020, Riedel's group presented the first report of complexes of the type [(SIMes)Au(CF_3_)_*x*_F_3‐*x*_] (*x=*1–3), **161–163**, prepared from treatment of [(SIMes)AuF_3_] **108** with TMSCF_3_ and CsF in DCM or THF (Figure 30C).^[54]^ In the presence of TMSCF_3_, selective exchange of the elongated *trans*‐fluoride with trifluoromethyl yielded *trans*‐[Au(CF_3_)F_2_(SIMes)] **161** and *cis*‐[Au(CF_3_)_2_F(SIMes)] **162** in a ratio of 7 : 1, respectively (in 0.5 equiv. TMSCF_3_). In THF, the complex was more soluble, and further substitution with all fluorides was observed (5 : 1 of **162** : **163**). Crystals of **161** were grown and the structure showed, expectedly, comparable lengths with **108**, (1.932 vs 1.916 Å respectively). Some decomposition of complexes to Au^(0)^ was reported, which is unexpected, considering the stability afforded by the NHC ligand.

### 4.4 N/C Multidentate Ligands

**Figure 31:**
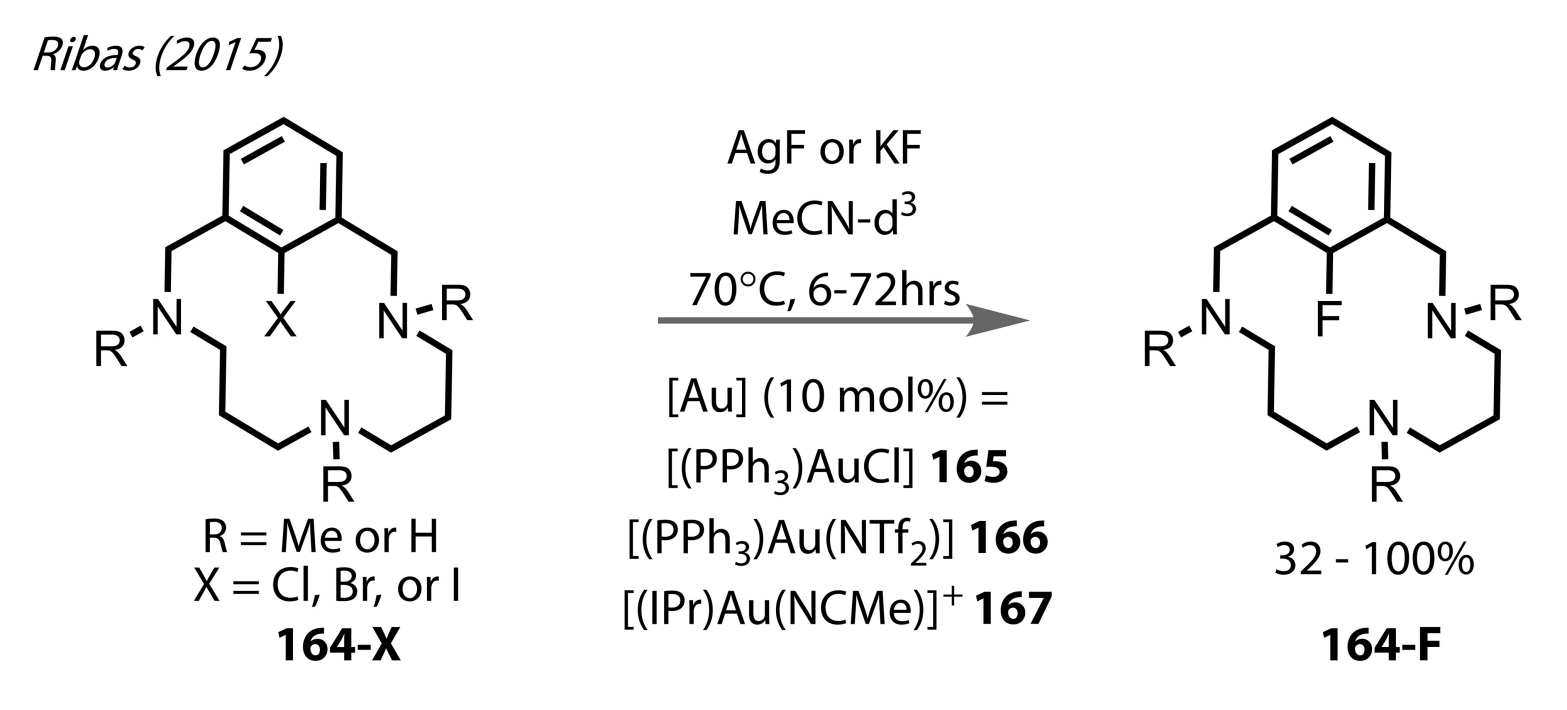
Ribas’ (2015) study on C−F reductive elimination via a putative Au^(III)^‐F intermediate, using various ligands (**165**–**167**) using a chelating aryl halide substrate **164‐X** to form the aryl fluoride **164‐F**.

In 2015, Ribas studied C−X reductive elimination proceeding through a putative Au‐X intermediate (X=F, Cl, Br, or I), using aryl halide‐tethered macrocycles **164‐X**, employing various gold catalysts (**165**–**167**) (Figure 31).^[102]^ This macrocyclic substrate has been instrumental in validating the existence of M^(I)^/M^(III)^ coupling mechanisms of Cu and Ag through chelation to the metal centre upon oxidative addition via the amine moieties. Especially high yields were achieved when reacting the aryl‐iodide macrocycle (X=I) bearing tertiary amine moieties (R=Me) with AgF (5 eq.). The authors did not describe the observation of an intermediate gold^(III)^‐fluoride complex, but is presumed to form in this reaction.

**Figure 32:**
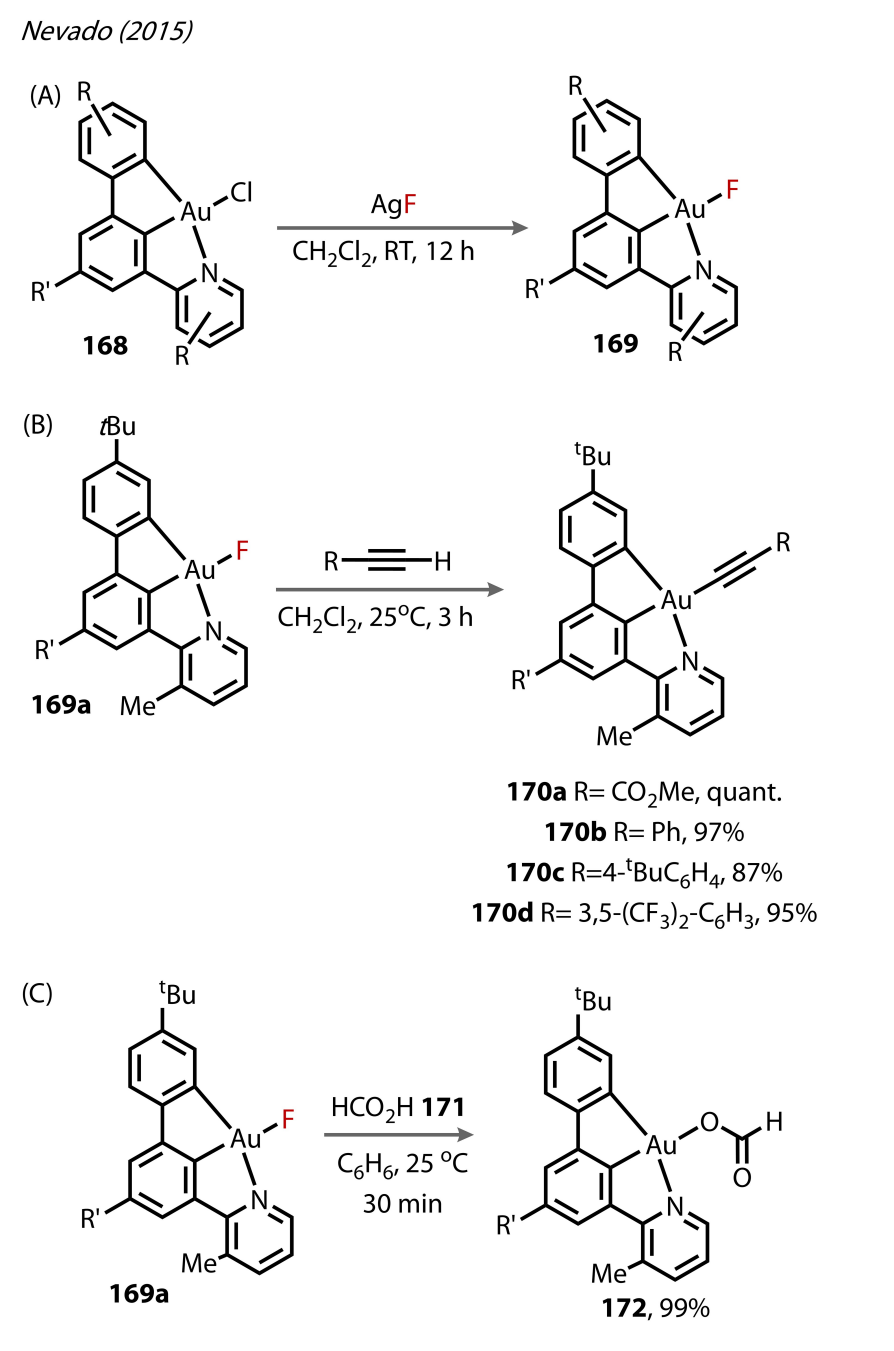
Nevado (2015). (A) Formation of [(N C C)AuF] **169** by Au‐Cl/Au‐F metathesis of **168** with AgF. Reactivity of [(N C C)AuF] **169** **a** (R’=4‐*t*‐BuC_6_H_4_) with (B) terminal alkynes to form Au‐alkynyl complexes **170** **a**–**d**, and (C) formic acid **171**.

Nevado was the first to use N C C ligands, reporting bench stable, monomeric *κ* ^*3*^‐[(N C C)AuF] (**169**) complexes in 2015 (Figure 32).^[101]^ The N C C framework was carefully designed such that the anionic carbon centres would stabilise electron‐poor Au^(III)^, whilst creating labile Au^(III)^‐Cl in **168**, due to the strong *trans* influence of the carbon ligand, so that the structure could be accessed from simple Cl/F exchange. Complexes **169** were prepared from the addition of AgF to *κ* ^*3*^‐[(N C C)AuCl] **168** in DCM (Figure 32A). The presence of Au^(III)^‐F bonds was confirmed by ^19^F NMR (δ resonances in the −230 to −225 ppm range in C_6_D_6_), and all complexes were found to be water and light stable for weeks in monomeric form. The reactivity of **169** **a** towards terminal alkynes was investigated (Figure 32B). **169** **a** was treated with four varying substituted alkynes, forming Au^(III)^‐alkynyl complexes in high yields (**170** **a**–**d**), with **170** **a** forming quantitatively. Finally, the Au^(III)^‐formate **172** could be accessed quantitatively through treatment with formic acid **171** (Figure 32C).^[103]^

**Figure 33:**
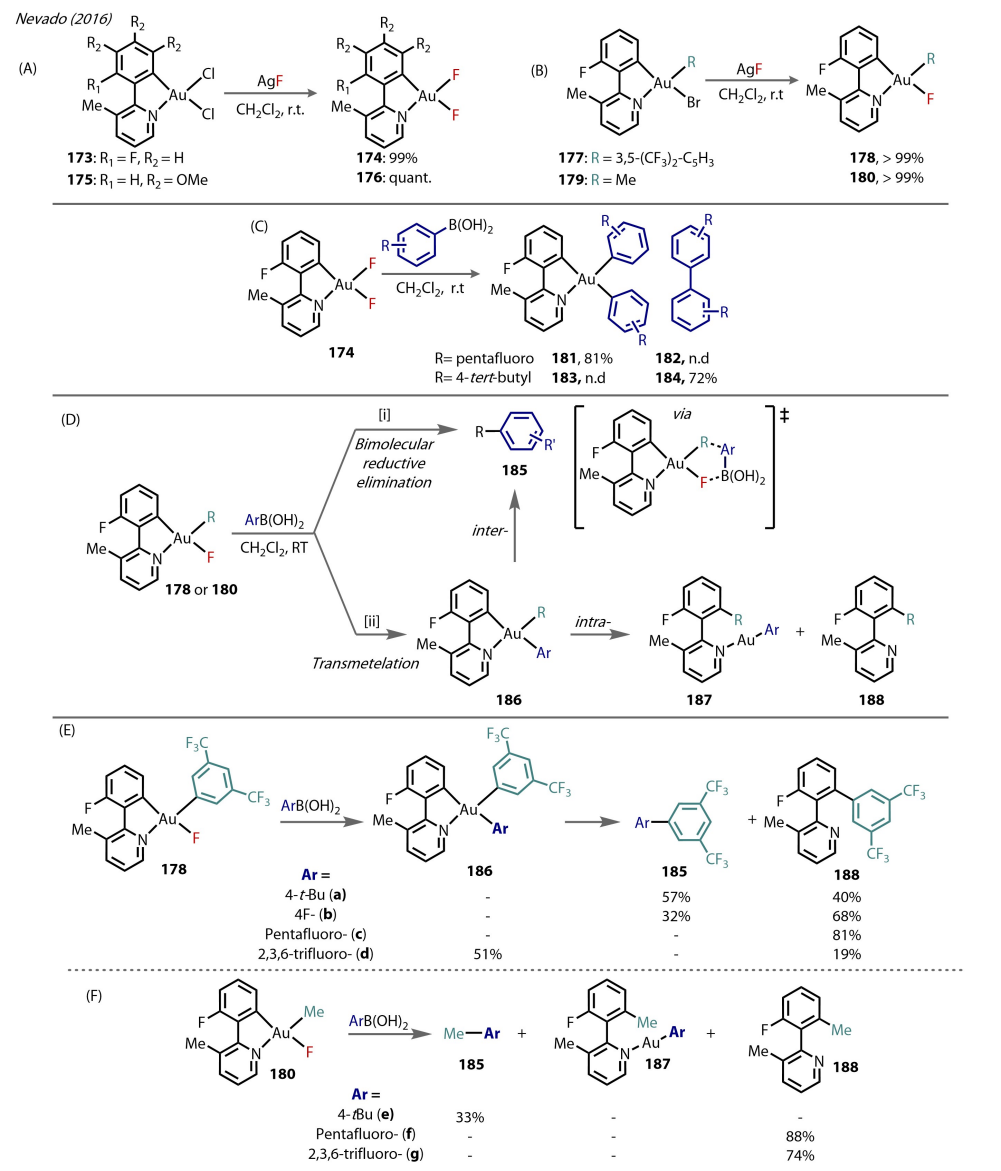
Nevado (2016). Synthesis of C N supported (A) Au‐difluoride (**174** and **176**), and (B) Au‐monofluoride **178** and **180** from halide ligand exchange with AgF of complex **177** and **179** respectively. (C) Homocoupling reactivity from the difluoride complex **174**. (D) Mechanistic pathways for cross coupling. with aryl‐boronic acids: [i] bimolecular reductive elimination to form **185**, [ii] transmetalation to form **186**, leading to **185** through reductive elimination with R, or **187/188** through reductive elimination onto the bidentate ligand. (E) Ar‐Ar‘ cross coupling study with **178** leading to biaryl coupling products **185** **a**–**b** or **188 (a**–**d)**. (F) Ar‐Me cross coupling study with **180** to form methylated arene products **185**, or **187/188** through C−C reductive elimination onto the bidentate ligand.

In 2016, Nevado reported the preparation of four monomeric Au^(III)^‐fluoride complexes with C N ligands (Figure 33), which show similar advantages to N C Cs.^[90]^ The complexes were prepared by treating 3‐methylpyridine‐Au^(III)^ complexes **173** and **175** with AgF to yield the corresponding Au^(III)^‐difluoride complexes, **174** and **176** (Figure 33A), which were all characterised by ^19^F NMR and X‐ray crystallography. A similar method was also used for Au^(III)^‐bromide complexes, **177** and **179**, which produced Au^(III)^‐monofluoride complexes of the type [(C N)Au(R)F], **178** and **180** (Figure 33B). Due to the greater *trans* effect of the aryl moiety, the *trans* Au‐F bond is elongated compared to that *trans* to the pyridino ligand for **174** (2.014 vs 1.951 Å) and **176** (2.025 vs 1.940 Å).

The reactivity of complex 174 towards homocoupling with various boronic acids was explored (Figure 32). **174** was treated with (pentafluorophenyl)boronic acid in DCM, producing the transmetalation product, [(C N)Au(C_6_F_5_)_2_], **181** exclusively, thus demonstrating that direct transmetalation was possible between boronic acids and (Csp^2^)‐Au^(III)^‐fluoride complexes. By contrast, reaction with (4‐*tert*‐butylphenyl)boronic acid yielded the *intermolecular* C−C coupling biaryl product **184**.

The heterocoupling reactivity of **178** and **180** with boronic acids was tested (Figure 33D) to provide insight into the two routes: bimolecular reductive elimination would yield just the *inter*molecular reductive elimination product, **185**, whereas direct transmetalation followed by either *intra*‐ or *inter*‐ molecular reductive elimination would yield **187/188** or **185**, respectively. For the reaction of **178** with 4‐(tertbutylphenyl)‐ and 4‐(fluorophenyl)boronic acids (Figure 33E), the presence of a mixture of the *inter‐*molecular (**185** **a**, **185** **b)** and *intra*‐molecular (**188** **a**, **188** **b**) products suggested the reaction proceeds via transmetalation and not via the bimolecular reductive elimination. **178** was then reacted with more electron‐deprived boronic acids; (pentafluorophenyl)‐ and (2,4,6‐ trifluorophenyl)‐boronic acid both produced the intramolecular product (**188** **c** and **188** **d**, respectively) in very good yields.

**Figure 34:**
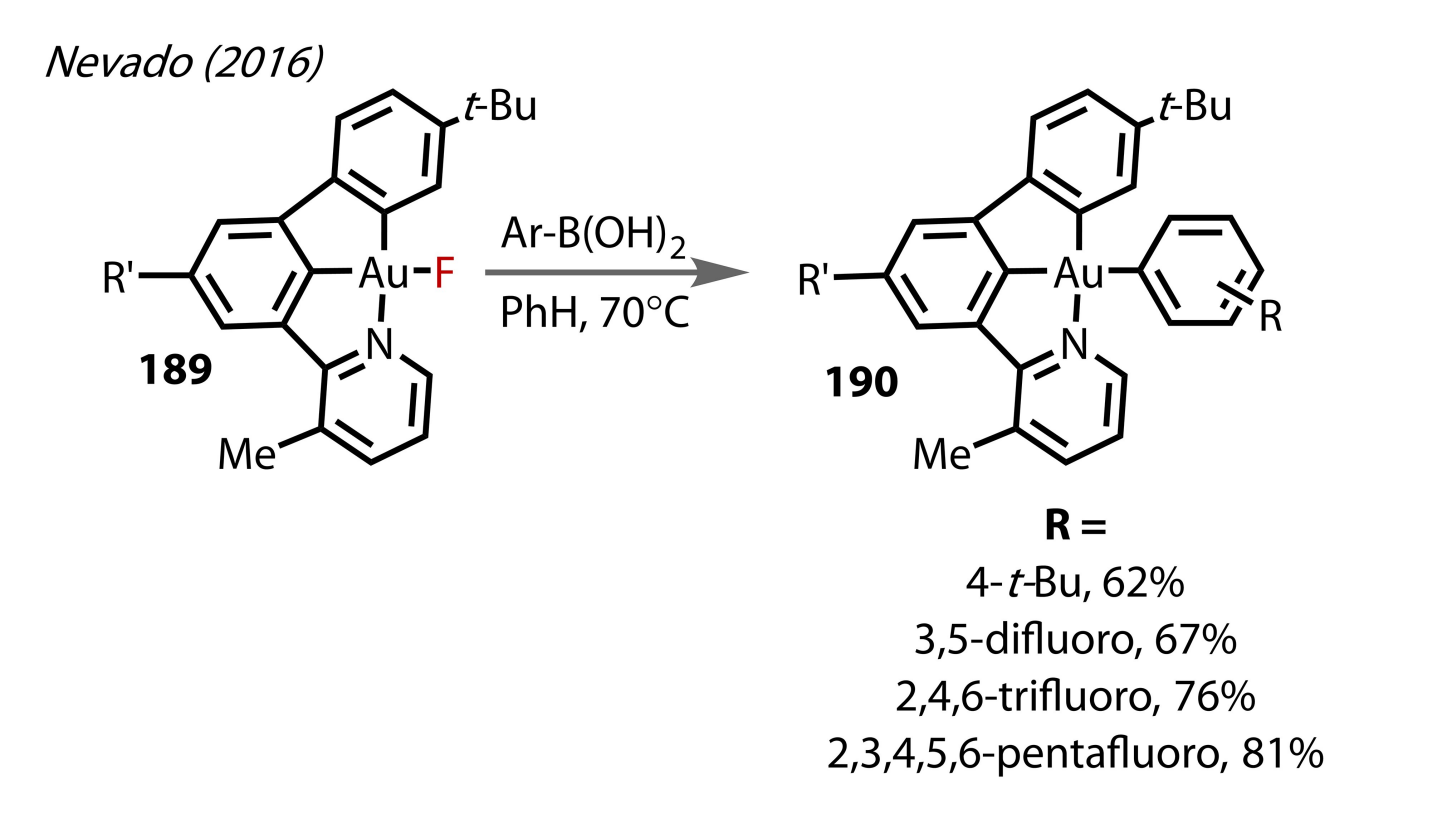
Nevado's (2016) C,C,N tridentate Au^(III)^‐F complex **189** (R′=4‐*t*‐BuC_6_H_4_−) undergoing direct transmetalation with arylboronic acids forming **190**.

Similar reactions were observed with **180** (Figure 33F). The use of 4‐(tertbutylphenyl)boronic acid yielded the inter‐ (**185** **e**) reductive elimination product, whereas only intra‐molecular reductive elimination (transferring the methyl group) was observed to give **188** **f/g** using electronically‐deprived boronic acids. It was clear from the range of products observed that both electronic and steric factors of the boronic acid influence the reaction pathway; unlike a bimolecular reductive elimination mechanism that is not typically influenced by electronic effects. The authors also observed direct transmetalation on the tridendate C,C,N complex **189** with various boronic acids to yield **190** (Figure 34), with most of the examples involving electron‐deprived boronic acids. These species were stable towards reductive elimination.

**Figure 35:**
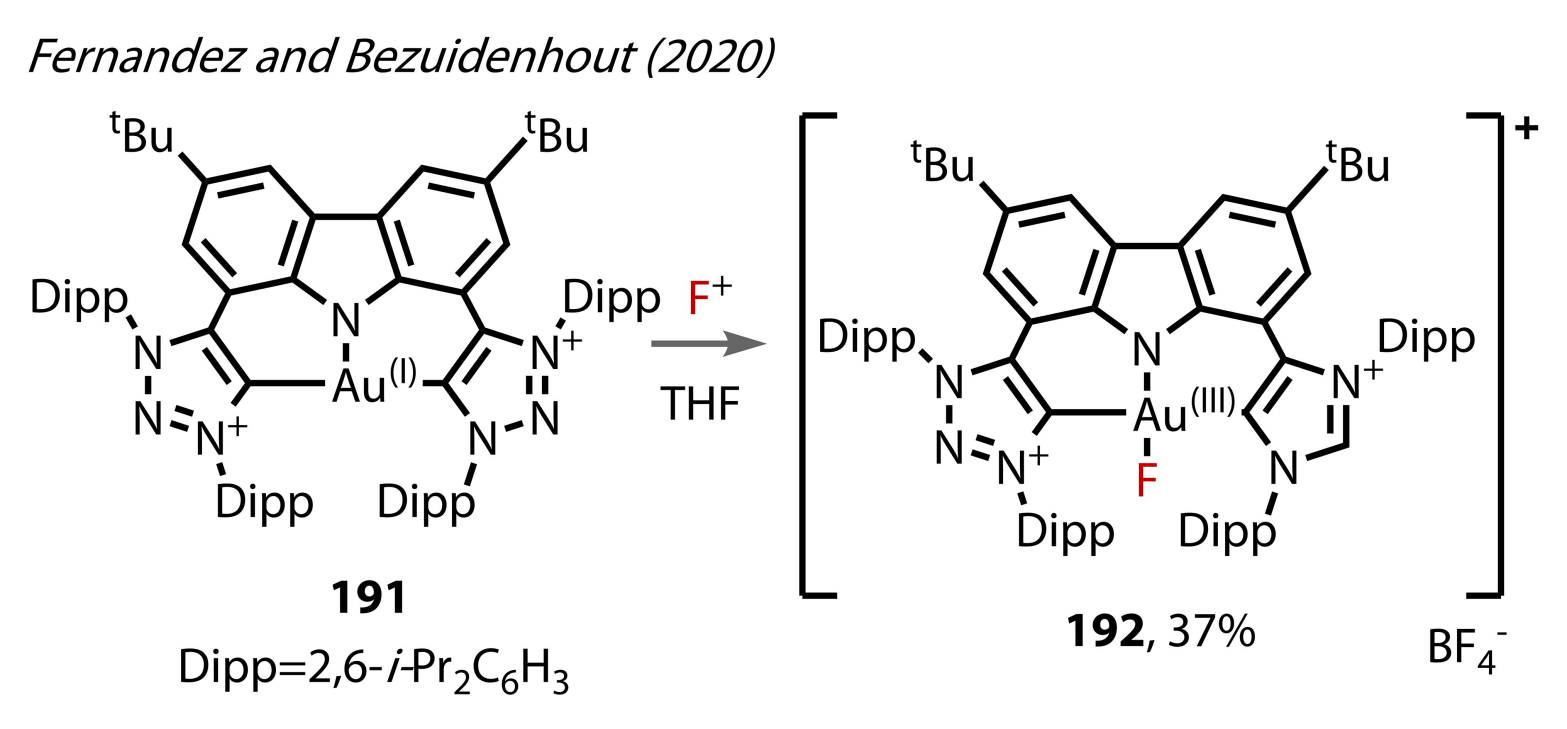
Fernandez and Bezuidenhout (2020): cationic C,N,C‐ligated Au^(III)^‐fluoride complex **192** accessed via selectfluor oxidation of **191**.

Fernandez and Bezuidenhout (2020) synthesised and isolated a C,N,C‐ligated Au^(III)^‐fluoride complex **192** (Figure 35), consisting of two 1,2,3‐triazol‐5‐ylidenes bound to a carbazolide, for photophysical, rather than reactivity, studies.^[104]^ The synthesis of **192** involved oxidation of the corresponding Au^(I)^ complex **191** with Selectfluor (Figure 35). The Au^(III)^‐F bond length in **192** was relatively short at 1.947 Å.

**Figure 36:**
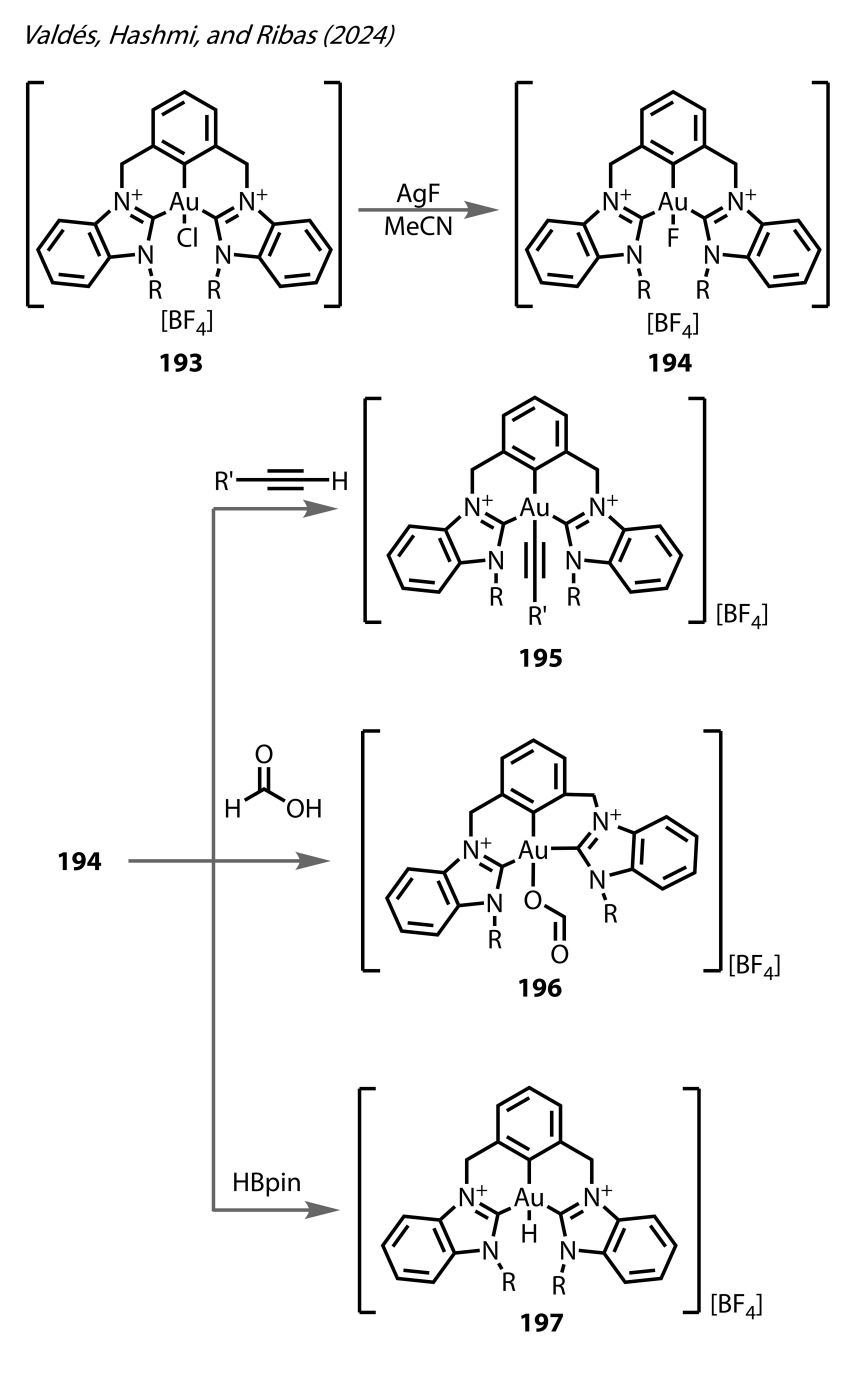
Valdés, Hashmi, and Ribas (2024): synthesis of C,C,C‐NHC‐Au^(III)^‐F pincer complex **194** from Cl/F substitution of **193** with AgF. The reactivity of **194** is demonstrated through alkynylation to **195**, acyloxylation to **196**, and H/F substitution with HBpin to form **197**.

In 2024, Valdés, Hashmi, and Ribas reported their explorations into various C,C,C‐NHC Au^(III)^ pincer complexes (Figure 36).^[105]^ The Au^(III)^‐Cl complex **193** underwent metathesis with AgF at room temperature to form the Au^(III)^‐F pincer complex **194**. The ^19^F NMR exhibited a signal at ~−260 ppm for both isopropyl and *n*‐butyl complexes, as expected, with minimal change in the ^1^H NMR. The carbene carbon of the isopropyl complex appeared as a doublet in the ^13^C{^1^H} NMR, arising from ^2^ *J* C−F coupling to the fluoride ligand (2.6 Hz at 170 ppm), with the Au‐C environment of the aryl ligand exhibiting a larger *trans* ^2^ *J* C−F of 27 Hz at 124.1 ppm. The basic nature of the fluoride ligand was examined in various reactions using Au^(III)^‐F complex. The complex readily underwent alkynylation with phenylacetylene and 1‐hexyne at room temperature to give **195**. Reaction with formic acid yielded the corresponding formate complex **196**, a species relevant to dehydrogenation reactions,[^106^, ^107^, ^108^] within minutes at room temperature. The formate complex was stable for several days at room temperature. To access the Au^(III)^‐H complex, the authors attempted to induce a β‐hydride elimination from the formate complex at elevated temperatures (in 1,2‐dichloroethane), but instead observed decomposition to the Au^(III)^‐Cl complex. As an alternative, the Au^(III)^‐F complex underwent transmetalation with H‐Bpin to form quantitatively the Au^(III)^‐H complex **197**.

### 4.5 Monodentate N‐Ligands

**Figure 37:**
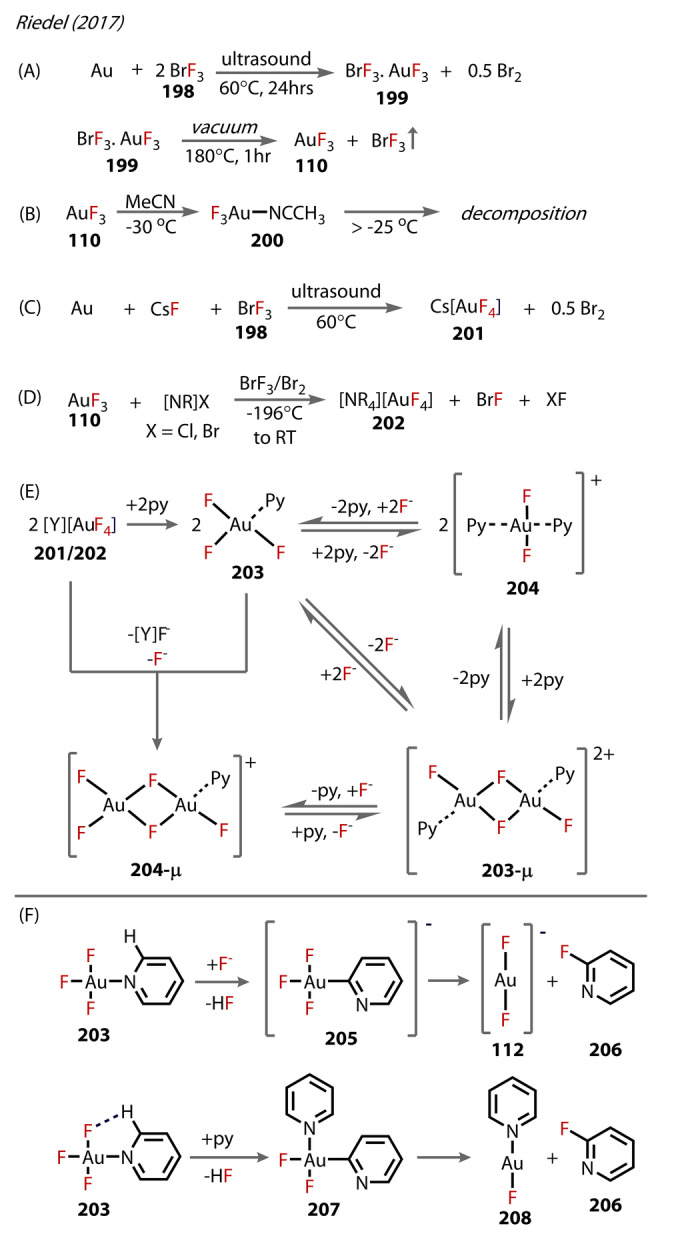
Riedel (2017). (A) Improved synthesis of AuF_3_.**110**. (B) Formation and decomposition of [(MeCN)AuF_3_] **200** at low temperatures. (C) Synthesis of Cs[AuF_4_] salt **201** using CsF in BrF_3_ solvent. (D) Synthesis of organic‐soluble [NR_4_][AuF_4_] (R=Me, Et) **202**. (E) Formation of mixture of pyridine adducts of Au^(III)^‐fluoride (**204** and **204**) from [Y][AuF_4_], where Y=Cs^+^ or NR_4_^+^ (R=Me or Et). (F) Possible routes to fluoropyridine **206** by intermolecular or intramolecular reductive elimination from **203**.

Pyridine and *N*‐imidazole ligands have long been known to stabilise Au^(III)^‐complexes.^[109]^ The first report of using these ligands to stabilise Au^(III)^‐fluoride complexes was in 2017 by Riedel and co‐workers (Figure 37).^[110]^ The authors first developed new routes to AuF_3_ and AuF_4_^−^ salts, particularly as the latter hold potential as convenient precursors to Au‐F complexes. Sonication of metallic gold in BrF_3_ **198** under heat yielded the BrF_3_.AuF_3_ **199** and Br_2_ as a by‐product. **199** is then heated under vacuum to remove the BrF_3_ (Figure 37A). The authors examined the behaviour of AuF_3_ **110** in various solvents, such as chloroform, DCM, and acetonitrile. In acetonitrile, the solvent‐coordinated complex [(NCCH_3_)AuF_3_] **200** (Figure 37B) formed, whereas in chlorinated solvents, Au^(III)^‐chloride species formed, as also observed in previous reports.[^48^, ^60^, ^74^] Although its formation was successful, NMR analysis showed that **200** was unstable, decomposing above −25 °C.

Regarding AuF_4_^−^ salts, previous methods require gaseous F_2_,^[111]^ or very dry conditions with (anhydrous) HF.^[112]^ Considering these drawbacks, the authors developed an improved procedure for synthesizing these aurate salts. [Cs]AuF_4_ **201** was prepared by sonicating Au^(0)^ with CsF in BrF_3_ at 60 °C (Figure 37C), revealing a ^19^F NMR shift of −305 ppm in MeCN. The salt was stable (~1 year) when stored in argon in a PFA tube and did not react with DCM (Cl/F exchange). Due to the very low solubility exhibited by **201**, the authors sought to prepare a more organic‐soluble AuF_4_ salt to examine complexation with various ligands. A variation of the previous method employs [NR_4_]X (R=Me, Et; X=Cl, Br) in a Br_2_/BrF_3_ mixture to obtain the [NR_4_][AuF_4_] salts **202** (Figure 37D), forming BrF and XF (X=Cl or Br) as by‐products. ^19^F NMR shifts of −304.1 ppm for R=Me, and −305.3 ppm for R=Et. The authors obtained crystal structures for [NR_4_][AuF_4_] salts **202** (R=Me and Et), which indicated Au‐F bond lengths in the range of 189.9 to 191.6 pm, and bond angles in the AuF_4_ anion varying from 88.5(6)° to 91.5(6)°, with no evidence of intermolecular Au‐F interactions (the shortest distance at 340 pm being longer than the sum of the van der Waals radii of 320 pm).

They detailed the synthesis of a mixture of pyridine‐supported Au^(III)^‐fluoride complexes that were prepared from the dissociation of fluoride from Cs[AuF_4_] **201** and [NMe_4_][AuF_4_] **202** salts in pyridine, resulting in a mixture of products (Figure 37E). The mono‐substituted complex, [(py)AuF_3_] (**203**) and its derivative **204** were observed by NMR, whilst the others, **203‐μ** and **204‐ μ**, were less stable and thus, only detected by ESI(+) spectroscopy. Due to the close spatial arrangement of the *ortho*‐H and fluoride on **204**, C−F reductive elimination occurred via **205**, yielding 2‐fluoropyridine **206** and HF, as observed by ^1^H‐ and ^19^F NMR (Figure 37F). The authors proposed two possible mechanisms for fluoropyridine formation (Figure 37F); an intermolecular route involving deprotonation of the *ortho*‐H by the basic fluoride followed by rearrangement to form **205**, or an intramolecular route with ligand‐assisted C−H activation, yielding a [(py)Au^I^F]) **208** by‐product .

**Figure 38:**
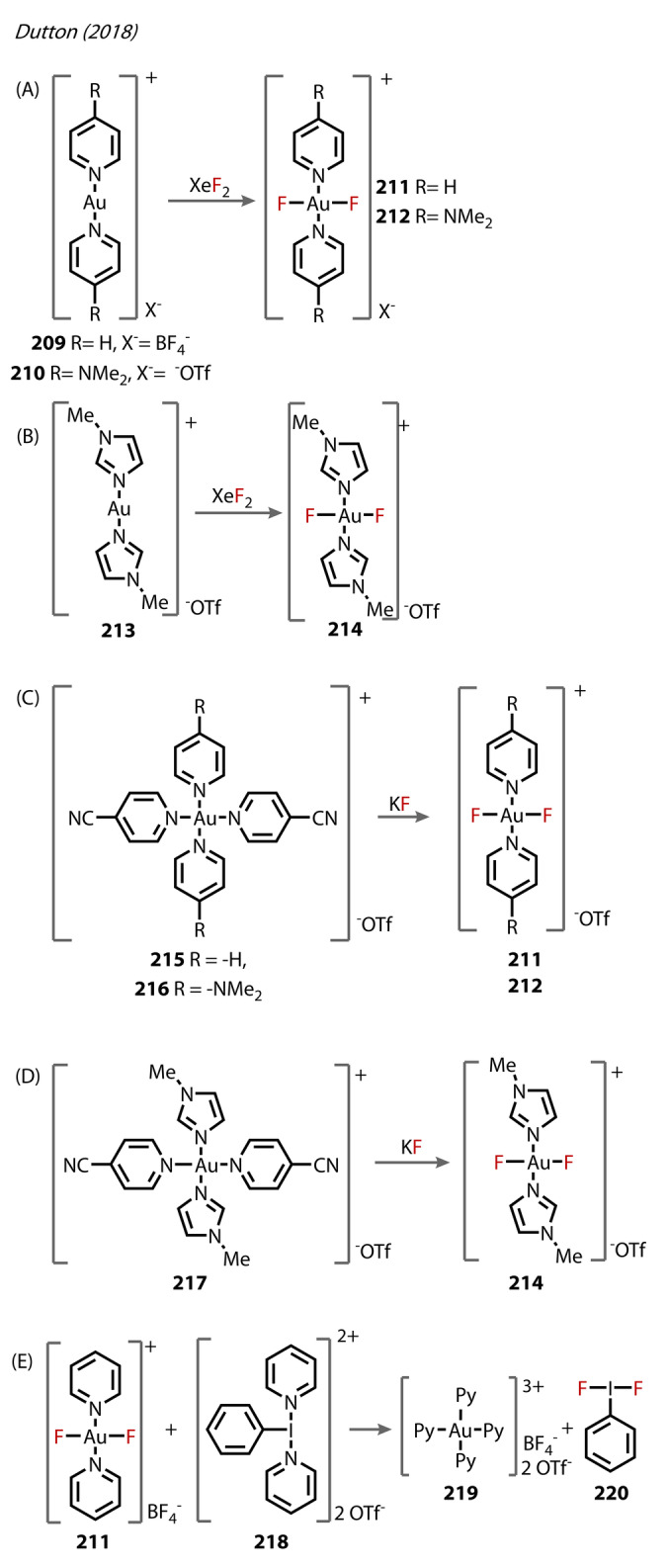
Dutton (2018) formation of (A) pyridyl (**211**, **212**) and (B) imidazolyl‐ *trans*‐Au^(III)^‐F_2_ complexes **214**. (C) **211** and **212** also form via fluoride displacement of pyridyl ligands *trans‐*to one another with KF from complexes **215/216**. (D) **217** undergoes the same substitution to form **214**. (E) Ligand exchange with a pyridine‐dicoordinated hypervalent iodine oxidant **218** to form [Au(py)_4_]^−^ **219**.

Dutton (2018) extended this work by investigating pyridine and *N*‐imidazole as supporting ligands for Au^(III)^‐fluorides,^[100]^ and, along with Menjón in the same year,^[99]^ reported the first isolated *trans*‐Au^(III)^‐difluoride complexes (Figure 38). The first of two synthetic methods involved oxidation of the Au^(I)^ precursor, [Au(I)(4‐DMAP)_2_][OTf] **210** and [Au(I)(py)_2_][BF_4_] **209** with XeF_2_ in chloroform to **211** and **212** (Figure 38A); both complexes were characterised by ^19^F NMR, but neither structure could be confirmed by X‐ray crystallography. The analogues with *N*‐methylimidazole ligands, **214** was prepared from **213** (Figure 38B) and were confirmed by X‐ray crystallography. KF was introduced as an alternative fluoride source, which delivered the desired Au^(III)^‐difluorides from reaction with [Au^III^(4‐cyanopyridine)_2_(R‐py)_2_][3OTf] **215** and **216** (Figure 38C) or [Au^III^(4‐cyanopyridine)_2_(N‐imidazole)_2_][3OTf] **217** (Figure 38D).

**Figure 39:**
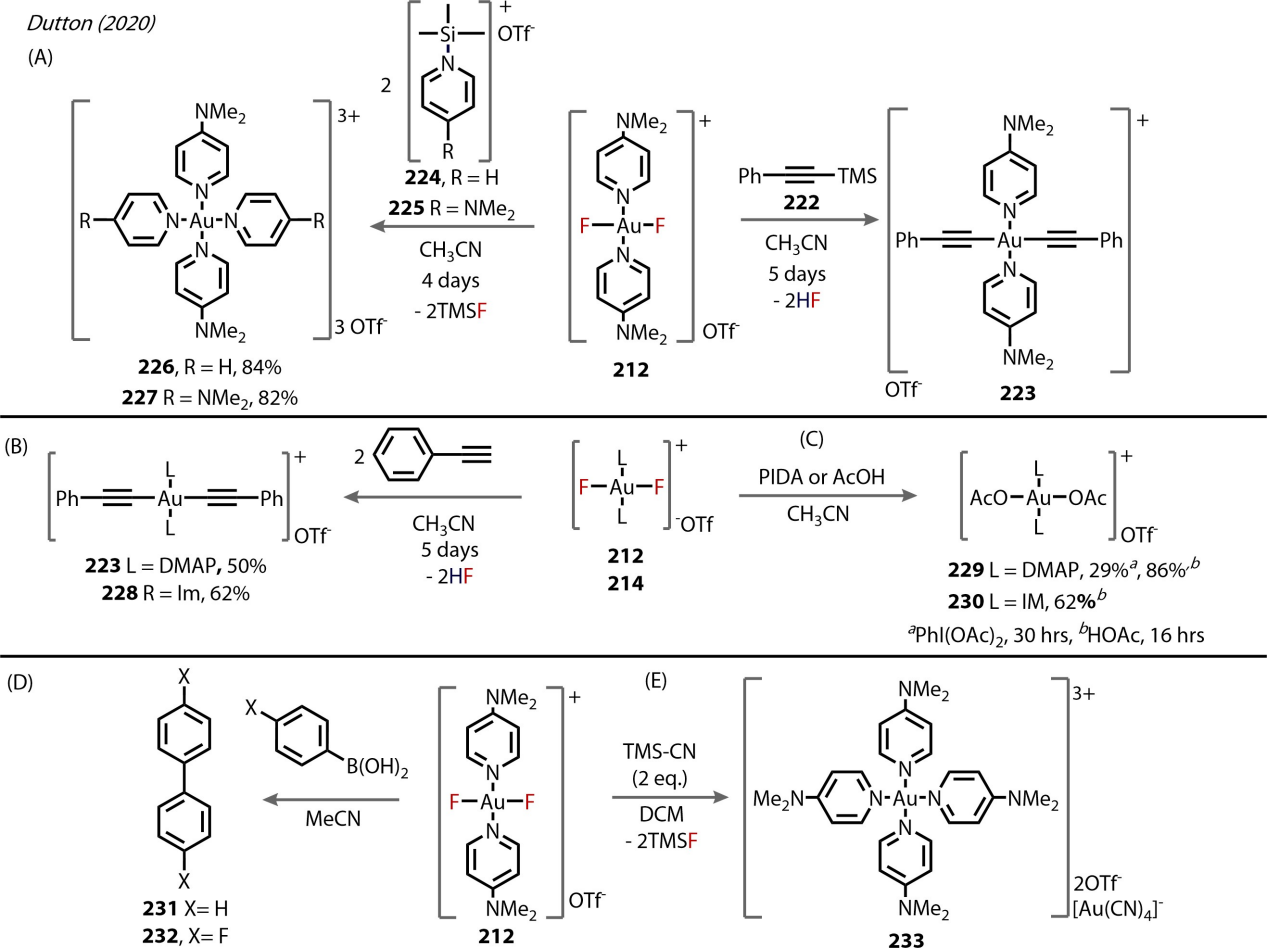
Dutton's (2020) reaction of *trans*‐difluoro gold complexes **212** and **214** with (A) TMS‐pyr reagents **224** and **225** and TMS‐CC(Ph) **222**, (B) Synthesis of *trans‐*alkynyl complex [Au{(L)(R)}_2_]^+^ **223** and **228** using H‐CC(Ph). (C) Synthesis of {Au[(L)(OAc)]_2_}^+^ **229** and **230**, using acetic acid or PIDA with [(L)AuF]^+^, where L=4‐dimethylamino pyridine or N‐methyl imidazole. (D) Reaction of **212** with phenylboronic acids, and (E) with [TMS‐CN] to form **233** instead of the expected F/CN substitution product.

The Au^(III)^‐F bond lengths for **214** were 1.908 and 1.924 Å, which are amongst the shortest Au^(III)^‐F bonds to be reported in an organometallic complex, and are comparable with terminal Au^(III)^‐F bond lengths in AuF_3_ (1.91 Å).^[113]^ With their high stability and their short Au^(III)^‐F bonds, it might be assumed they would be relatively inert. However, **211** readily underwent ligand exchange with [PhI(py)_2_]^2+^ **218**, to generate **219** and PhIF_2_ **220** (Figure 38E).^[114]^ **212** was also reacted with trimethyl(phenylethynyl)silane **222** (Figure 39A), which were of interest given the luminescent properties of Au^(III)^‐acetylides. The higher *trans*‐influence of the acetylide relative to fluoride facilitates rapid addition of the second acetylide, forming **223**. Metathesis reactions of both pyridine and N‐imidazole cationic Au^(III)^‐fluoride complexes were studied by Dutton and co‐workers.^[115]^ Au^(III)^‐fluoride **212** reacted with TMS‐py reagents **224** and **225** (Figure 39A), forming the cationic pyridyl‐coordinated **226** and **227** over four days in very good yield (^19^F NMR ) for both R groups. Phenylacetylene also readily undergoes alkynylation (Figure 39B), forming **223** and **228** from **212** and **214**, respectively.

After the success of the pyridine‐supported Au^(III)^‐fluorides undergoing metathesis reactions with I^(III)^ centres in the previous study,^[114]^ the authors attempted to investigate this using PIDA and observed the desired complex, **229** (Figure 39C). Likewise, **229**, which could not be synthesised from **210** and PIDA, was accessible in very good yields by employing acetic acid.

The reactivity of the Au^(III)^‐difluoride complex **212** with boronic acids was investigated. As previously observed,[^76^, ^90^] the reactivity of Au^(III)^‐fluorides with coupling partners has been extensively studied for weakly bound and, therefore, more reactive Au^(III)^‐fluorides, but has not been extended yet to strongly bound Au^(III)^‐fluorides. The difluoride complex **212** was reacted with aryl‐boronic acids to form the biaryl coupling products **231** and **232** (Figure 39D). When attempting to exchange the fluoride ligands on **212** with CN using TMS‐CN, **233** was observed (Figure 39E), reportedly from rapid subsequent ligand exchange of the desired Au^(III)^‐CN complex.

## 5 Conclusion

Promising advances have been made in the synthesis and characterisation of Au‐fluoride complexes. These complexes serve as catalysts or intermediates in a family of fluorination reactions, and hence a greater understanding of them is a central goal for the development of new transformations. The isolation and study of Au‐fluoride complexes remains a challenging task, but, as an emerging and exciting field, there is much to be explored, with applications extending to many domains of organic synthesis and catalysis.

Au^(I)^‐fluoride complexes are most notably reported with the use of Buchwald‐type phosphine ligands, which have enabled the study of various hydrofluorination and transmetalation pathways. Furthermore, reports of bridging Au^(I)^‐F complexes, though few, imply the relevance of such intermediates in C−F bond formation reactions. Fundamental studies on these species are rare, and should benefit this field.

The lack of Au^(II)^‐fluoride complexes is surprising considering their potential applications in catalysis leaving a clear gap in the literature for future fluorination reactions employing such species.

By contrast, many reports of Au^(III)^‐fluoride complexes exist, where multidentate ligand frameworks confer high stability. C N and N C C ligand supported complexes have been beneficial in regards to their reactivity with boronic acids, giving evidence that Au^(III)^‐fluorides could undergo a conventional transmetalation /reductive elimination sequence. Stoichiometric studies on Au^(III)^‐fluorides have been instrumental in validating the transmetalation mechanism with a diverse array of coupling partners. The precedent for electron donating, bidentate P N ligands, along with the ability of gold to mediate hydrofluorination reactions, present clear opportunities for the development of organic methods involving Au^(I)^/Au^(III)^ catalysis, particularly for C−F, C−C bond formation.

## Conflict of Interests

The authors declare no conflict of interest.

6

## Biographical Information

*Alexi T. Sedikides obtained his Ph.D. at the University of Bristol in 2023, under the guidance of Dr. Alastair J. J. Lennox. He is currently a postoctoral research associate in the same group, developing synthetic methodologies towards organofluorine motifs*.

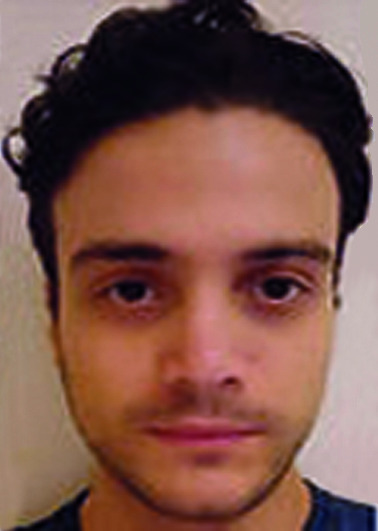

## Biographical Information

*Rhian C. Walters graduated from the University of Bristol with an M.Sc. in Chemistry with a study abroad in 2021. She then worked as a Technology consultant at KPMG before moving to Aston Martin as a Sustainability Advisor*.

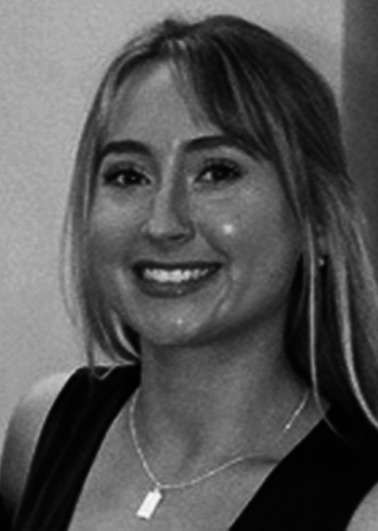

## Biographical Information

*Alice C. Dean received her Ph.D. in 2024 from the Technology Enhanced Chemical Synthesis Centre for Doctoral Training at the University of Bristol working under the supervision of Dr Alastair Lennox. She worked on hypervalent iodine mediated fluorination reactions. Alice now works in medical writing*.

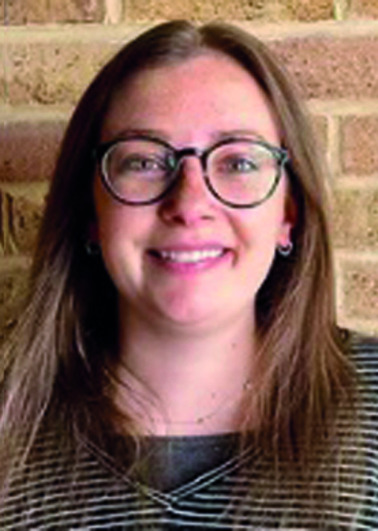

## Biographical Information

*Alastair J. J. Lennox attained his Ph.D. from the University of Bristol (Prof. Guy Lloyd‐Jones) and did postdoc studies in Rostock, Germany (Prof. Matthias Beller) as an Alexander von Humboldt Fellows and at the University of Wisconsin, Madison (Prof. Shannon Stahl). In 2018, Alastair returned to the University of Bristol as a Royal Society University Research Fellow to start his independent research programme. He was promoted to Associate Professor of Chemistry in 2022. His group are interested in the development of novel synthetic organic methods with sustainability and mechanism as themes that strongly underpin their approach to this. Specific interests include the exploration of electrochemistry as a tool for performing selective redox transformations, and also in the development of fluorination reactions and fluorinated building blocks*.

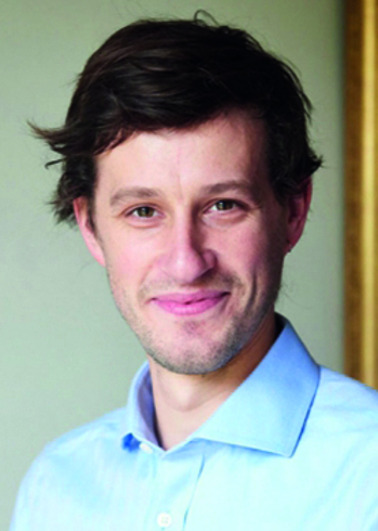

## Data Availability

Data sharing is not applicable to this article as no new data were created or analyzed in this study.
